# The protozoan commensal *Tritrichomonas musculis* is a natural adjuvant for mucosal IgA

**DOI:** 10.1084/jem.20221727

**Published:** 2024-11-13

**Authors:** Eric Yixiao Cao, Kyle Burrows, Pailin Chiaranunt, Ana Popovic, Xueyang Zhou, Cong Xie, Ayushi Thakur, Graham Britton, Matthew Spindler, Louis Ngai, Siu Ling Tai, Dragos Cristian Dasoveanu, Albert Nguyen, Jeremiah J. Faith, John Parkinson, Jennifer L. Gommerman, Arthur Mortha

**Affiliations:** 1 Department of Immunology, University of Toronto, Toronto, Canada; 2 Department of Biochemistry, University of Toronto, Toronto, Canada; 3 Peter Gilgan Centre for Research and Learning, The Hospital for Sick Children, Toronto, Canada; 4 Precision Immunology Institute, Icahn School of Medicine at Mount Sinai, New York, NY, USA; 5 Icahn Institute for Data Science and Genomic Technology, Icahn School of Medicine at Mount Sinai, New York, NY, USA; 6 Department of Molecular Genetics, University of Toronto, Toronto, Canada

## Abstract

Immunoglobulin (Ig) A supports mucosal immune homeostasis and host–microbiota interactions. While commensal bacteria are known for their ability to promote IgA, the role of non-bacterial commensal microbes in the induction of IgA remains elusive. Here, we demonstrate that permanent colonization with the protozoan commensal *Tritrichomonas musculis* (*T.mu*) promotes T cell–dependent, IgA class-switch recombination, and intestinal accumulation of IgA-secreting plasma cells (PC). *T.mu* colonization specifically drives the expansion of T follicular helper cells and a unique ICOS^+^ non-Tfh cell population, accompanied by an increase in germinal center B cells. Blockade of ICOS:ICOSL co-stimulation or MHCII-expression on B cells is central for the induction of IgA following colonization by *T.mu*, implicating a previously underappreciated mode of IgA induction following protozoan commensal colonization. Finally, *T.mu* further improves the induction of IgA-secreting PC specific to orally ingested antigens and their peripheral dissemination, identifying *T*.*mu* as a “natural adjuvant” for IgA. Collectively, these findings propose a protozoa-driven mode of IgA induction to support intestinal immune homeostasis.

## Teaser

The gut commensal protist *Tritrichomonas musculis* (*T.mu*) boosts T cell–dependent IgA. *T.mu*-driven IgA modifies the commensal bacterial microbiota and improves the generation and dissemination of IgA-secreting plasma cells reactive against orally ingested food antigens.

## Graphical Abstract

**Figure:**
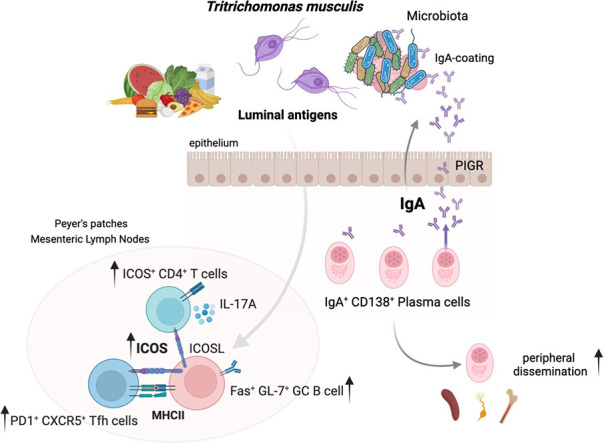

## Introduction

The intestinal microbiota encompasses a diverse collection of microorganisms inclusive of viruses, bacteria, fungi, worms, and protozoa (Blander et al., 2017). While bacteria, fungi, worms, and viruses have been recognized as regulators of the immune system, the contribution of commensal protozoan members of the microbiome remains less well understood (Cao and Mortha, 2020). Recent reports characterized the murine commensal protozoa, *Tritrichomonas musculis* (*T*.*mu*), *Tritrichomonas muris* (*T*.*muris*), and *Tritrichomonas rainier* (*T*.*rainier*) as agents of mucosal immune modulation (Howitt et al., 2016; Nadjsombati et al., 2018; Chudnovskiy et al., 2016; Escalante et al., 2016). *Tritrichomonas* spp. have been shown to contribute to the regulation of the T helper (h)1 and Th17 responses with beneficial and detrimental outcomes on the immunity and anti-microbial defense respectively (Chiaranunt et al., 2022; Chudnovskiy et al., 2016). Moreover, *Tritrichomonas* spp drives a tuft cell–group 2 innate lymphoid cells (ILC2) circuitry through microbial succinate to support anti-helminth immunity and epithelial regeneration (Schneider et al., 2018). Surprisingly, colonization by *T*.*mu* was found to ameliorate central nervous inflammation in a mouse model of experimental autoimmune encephalomyelitis by promoting the accumulation of gut-derived plasma cells (PC) in the brain, collectively highlighting the multifactorial contributions of *Tritrichomonas* spp. in modulating host tissue and immune homeostasis (Rojas et al., 2019).

Intestinal homeostasis requires interactions between the gut immune system and the commensal microbiota (Belkaid and Hand, 2014). IgA, the most prevalent isotype at mucosal surfaces, plays an essential role in maintaining the mucosal barrier, regulating bacterial growth, gene expression, and host immunity to oral antigens (Cerutti et al., 2011). IgA exists in monomeric, dimeric, and secreted forms, the latter, termed secretory IgA (SIgA). SIgA is comprised of an IgA-dimer joined by a J-chain and the secretory component (SC), a polypeptide that stabilizes the Fc regions of IgA dimers. This complex forms following the successful transportation of dimeric IgA across the intestinal epithelium via the polymeric-Ig receptor (pIgR) (Johansen and Kaetzel, 2011). Secreted into the lumen, SIgA binds to intestinal microbes and luminal antigens to mediate barrier support and homeostatic functions (Brandtzaeg and Prydz, 1984; Brandtzaeg, 2009). The intestinal lamina propria (LP) contains the largest population of IgA-secreting PC, which develops through T cell–dependent (Td) and T cell-independent (Ti) class switch recombination (CSR). This process mediates the differentiation of naïve B220^+^IgM^+^ B cells into IgA-secreting PC in gut-associated lymphoid tissue (GALT), including Peyer’s patches (PP), mesenteric lymph nodes (MLN), and isolated lymphoid follicles (ILF) (Isho et al., 2021; Fadlallah et al., 2019). During Ti IgA CSR, reactive oxygen species (ROS), retinoic acid (RA), tumor necrosis factor α (TNFα), transforming growth factor beta (TGFβ), interleukin (IL)-5, IL-6, IL-21, and tumor necrosis factor ligand superfamily members 13 (*TNFSF13*) and 13 b (*TNFSF13B*, *BAFF*) increase chromatin accessibility of the IgA α locus in antigen-receptor stimulated B cells (Cerutti, 2008) In addition to antigen-receptor stimulation, Ti IgA CSR can be mediated via the stimulation of toll-like receptors (TLRs) on B cells (Li et al., 2019). Conversely, Td IgA CSR requires the interaction of germinal center (GC) B cells with CD4^+^ T follicular helper (fh) cells that regulate the production of IgA through T cell antigen receptor/MHCII interaction and costimulatory receptor-ligand pairs CD40^−^CD40L or ICOS-ICOSL (Fagarasan et al., 2010). Td IgA CSR is believed to depend on antigen presentation by dendritic cells (DC) and follicular dendritic cells (FDC), providing a template for the selection of high-affinity GC B cells to generate antibody-secreting PC (Macpherson and Uhr, 2004; Tezuka and Ohteki, 2019; Hou et al., 2014). Both pathways lead to the differentiation of GC B cells into PC and result in the migration of IgA-secreting PC into the LP. However, clear differences in affinity and reactivity against commensal microbes have been reported when comparing either Ti and Td IgA CSR in mice (Bunker et al., 2015). This suggests that selective engagement of these pathways shapes the reactivity of IgA toward commensal microbes. The generation of IgA within the gut is supported by the gut microbiota and is readily able to adapt to new microbial members colonizing the intestinal tract (Li et al., 2020). Interestingly, once reactivity against gut bacteria has been established, the reactivity persists even if the colonization by the targeted microbe is transient (Hapfelmeier et al., 2010). Moreover, IgA-specificity against intestinal bacteria has been shown to identify disease-promoting bacteria in Crohn’s disease patients supporting the notion that IgA serves as barrier-sustaining agent for intestinal homeostasis (Palm et al., 2014).

However, the composition of the intestinal microbiota is not limited to bacteria, and the production and generation of IgA-secreting PC driven by non-bacterial microorganisms serves as a putative additional determinant of intestinal homeostasis (Cao and Mortha, 2020). Gut protozoa, such as *Tritrichomonas* are an underappreciated component of the intestinal microbiota even in humans, working across many yet-to-be-identified mechanisms that support the adaptive immune system including the activation of IgA-secreting PC. We thus hypothesized that intestinal protozoa can regulate the mucosal IgA response.

In this study, we report that the murine protozoa *T.mu* regulates both the intestinal and systemic IgA responses and uncover the cellular and molecular mechanisms involved in *T.mu*-driven IgA CSR, host–microbiome interactions, and mucosal homeostasis. Our findings show that colonization by *T*.*mu* increases luminal IgA levels and CD138^+^IgA^+^ PC counts, accompanied by higher numbers of PD-1^+^CXCR5^+^ Tfh cells and GL-7^+^FAS^+^ GC B cells in GALT. IgA induction following protozoan colonization requires Td IgA CSR in an ICOS:ICOSL and MHCII-TCR-dependent fashion. Subsequent analysis of the 16S rDNA gene sequences of IgA-coated gut bacteria indicates that *T*.*mu* colonization shifts the antibacterial IgA reactivity. Moreover, the elevation of IgA after colonization with *T*.*mu* improved the induction and peripheral dissemination of IgA-secreting PC specific to orally ingested food antigens. Our data collectively demonstrate that *T*.*mu* colonization acts as a natural adjuvant for IgA by potentiating the mucosal and systemic IgA response.

## Results

### Increased IgA levels following colonization with *T*.*mu*

**Figure 1.**
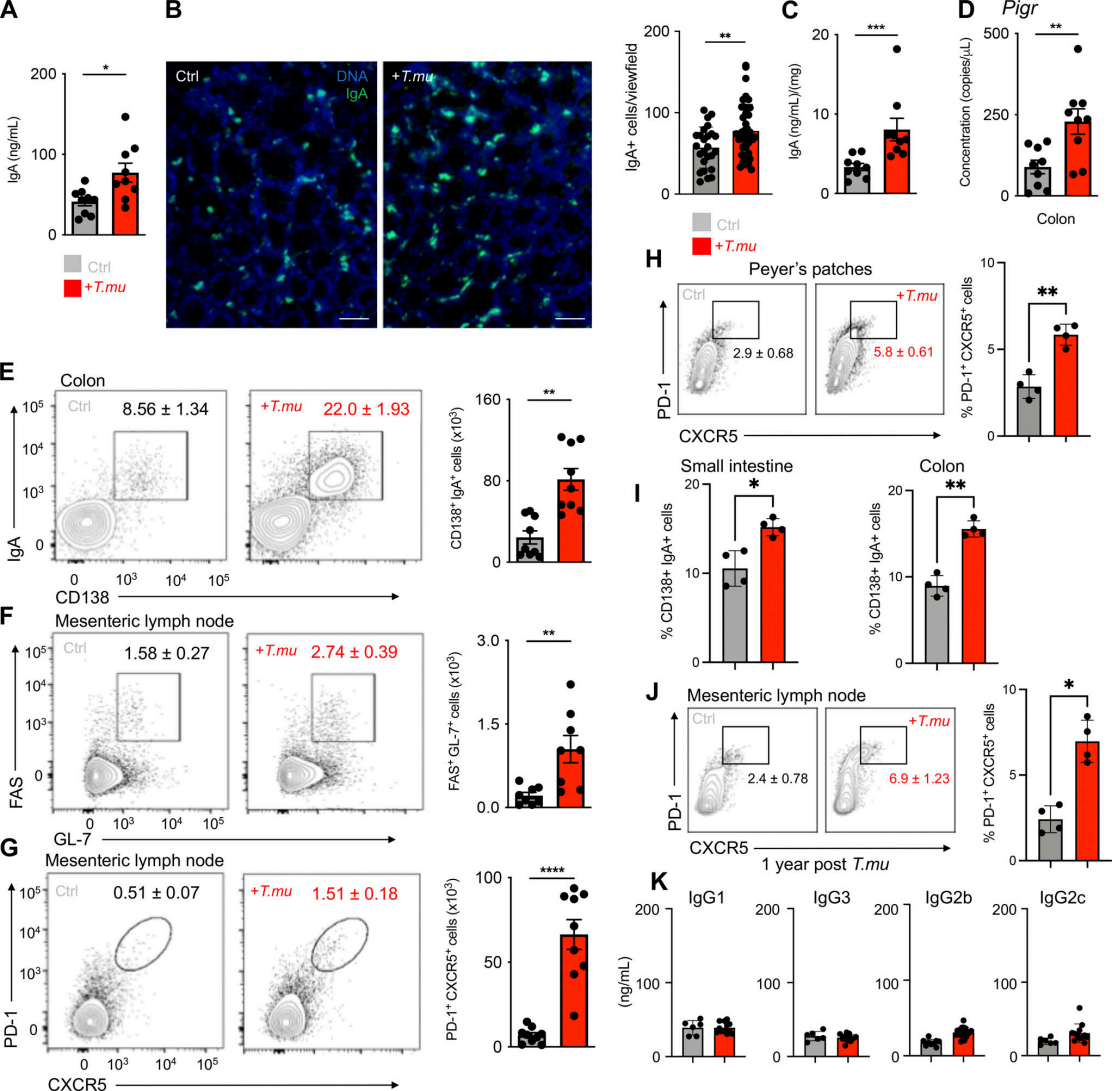
***T***.***mu* induces mucosal IgA. (A)** Serum IgA levels in control and *T.mu* colonized mice. **(B)** Representative immunofluorescence images of colonic tissue sections from control (left) and *T.mu* colonized mice (right) samples. Sections were stained with anti-IgA antibodies. Adjacent bar graph shows the quantification of IgA^+^ cells. **(C)** IgA levels in fecal samples. **(D)** Gene expression of *Pigr* in whole colonic tissue. **(E)** Representative contour plots of colonic lamina propria leukocytes stained for IgA and CD138. Gates identify IgA^+^CD138^+^ PC. **(F)** Contour plots show FAS^+^GL-7^+^ GC B cells in the MLN of control or *T.mu* colonized mice. **(G)** Representative flow cytometry plots identifying PD-1^+^CXCR5^+^ Tfh cells in the MLN of control or *T.mu* colonized mice. Numbers adjacent to gates show percentages ± SEM in E–G. Bar graphs adjacent to E–G show absolute numbers of the indicated cell type ± SEM. **(H)** Representative flow cytometry plots identifying PD-1^+^CXCR5^+^ Tfh cells in the PP of control or *T.mu* colonized mice. Numbers adjacent to gates show percentages ± SEM and the adjacent bar graph indicates quantification across two independent experiments. **(I)** Bar graphs show percentages of small and large intestinal IgA^+^CD138^+^ PC in control and *T.mu* colonized mice. **(J)** Representative flow cytometry plots identifying PD-1^+^CXCR5^+^ Tfh cells in the MLN on control mice or animals colonized with *T.mu* for 1 year. Numbers adjacent to gates show percentages ± SEM and adjacent bar graph indicates quantification across two independent experiments. **(K)** Bar graphs indicate concentrations of IgG isotypes in the serum of control or *T.mu* colonized mice. Data from at least two to three independent experiments with two to four mice/group are shown. Mann–Whitney *U* test was performed. * = P ≤ 0.05, ** = P < 0.01, *** = P < 0.001, **** = P < 0.0001, NS = not significant. If not stated otherwise, displayed values include ± SEM.

**Figure S1.**
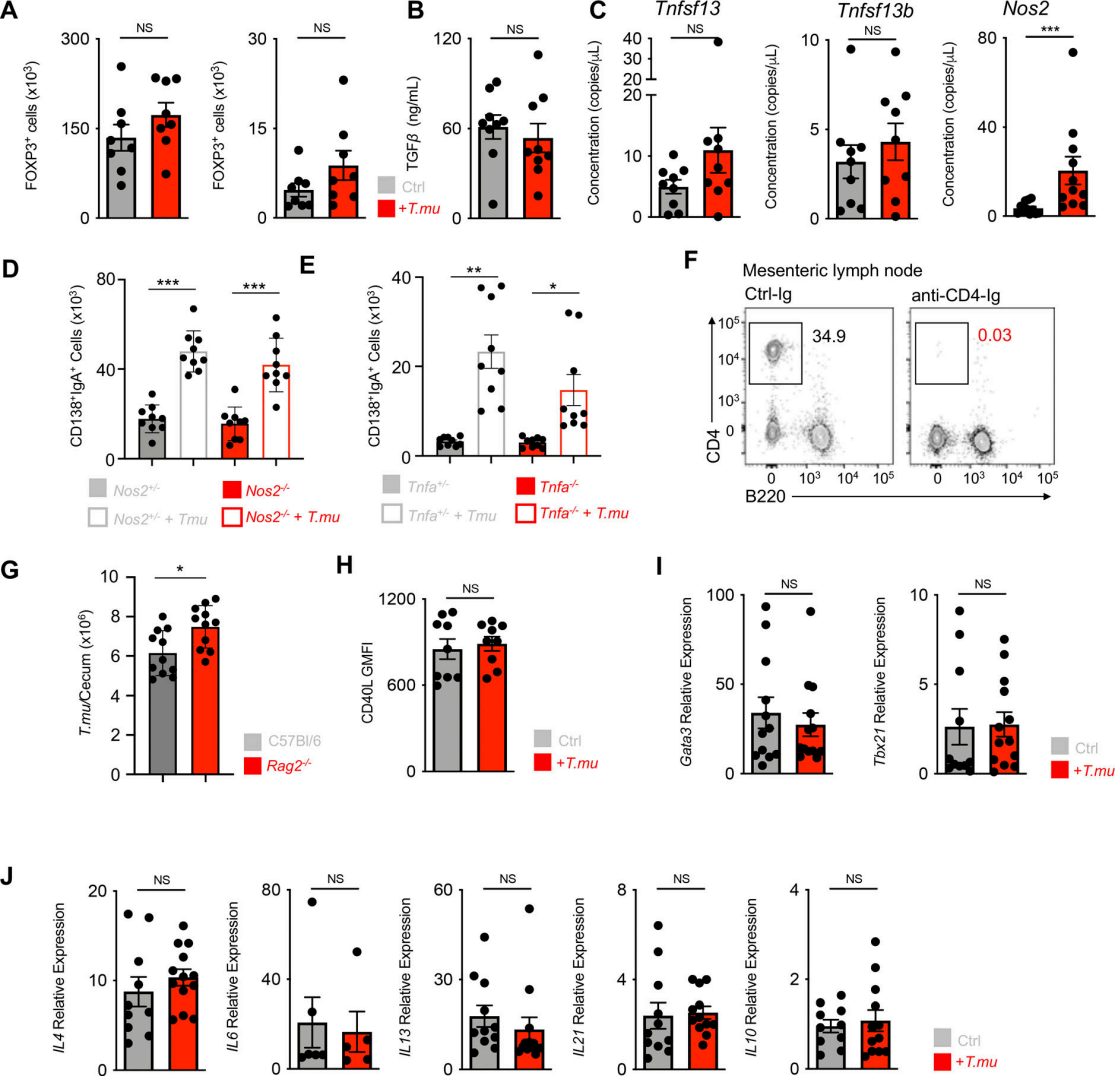
**Unaltered Tfh cell phenotype in the presence of *T.mu*. (A)** Quantification of FOXP3-expressing CD4^+^ T cells in the colonic lamina propria and MLN of control and *T.mu* colonized mice. **(B)** Quantification of bioactive TGF-β in colonic tissue explants from control and *T.mu* colonized mice. **(C)** Gene expression analysis of *Tnfsf13*, *Tnfsf13b*, and *Nos2* in total colonic tissue. **(D)** *Nos2*^*+/−*^ and *Nos2*^*−/−*^ mice were either left untreated or were colonized with *T.mu*. IgA^+^CD138^+^ PC were quantified in the colonic lamina propria. **(E)** *Tnfa*^*+/−*^ and *Tnfa*^*−/−*^ mice were either left untreated or were colonized with *T.mu*. IgA^+^CD138^+^ PC were quantified in the colonic lamina propria. **(F)** Representative staining for CD4 on lymphocytes in the blood of mice treated with anti-CD4-Ig. **(G)** Absolute numbers of *T.mu* from the caecum of C57BL/6 and *Rag2*^*−/−*^ mice 3 wk after oral gavage with 2 × 10^6^ FACS-sorted *T.mu*. **(H)** CD40L expression on MLN PD-1^+^CXCR5^+^ Tfh cells from control or *T.mu* colonized mice. **(I and J)** PD-1^+^CXCR5^+^ Tfh cells were sorted from the MLN of control or *T.mu* colonized mice. Gene expression analysis was performed for (I) transcription factors *Gata3* and *Tbx21* and (J) cytokines *Il4*, *Il6*, *Il13*, *Il21*, *Il10*. Data shown are representative of at least two to three independent experiments with three to four mice/group. Mann–Whitney *U* test was performed. * = P ≤ 0.05, ** = P < 0.01, *** = P < 0.001, **** = P < 0.0001, NS = not significant. If not stated otherwise, displayed values include ± SEM.

Mice inoculated with the intestinal commensal protozoa *T.mu* show life-long colonization and do not develop signs of spontaneous inflammation and pathology (Chudnovskiy et al., 2016). Previous reports demonstrated that colonization of the intestinal tract by newly colonizing gut bacteria impacts the mucosal IgA response (Hapfelmeier et al., 2010). We therefore investigated whether colonization by *T.mu*, as a non-bacterial commensal, could similarly change the mucosal IgA response. We first sought to determine serum IgA antibody levels of mice orally colonized by *T.mu* after 21 days, a time point that enables stable engraftment and sufficient time for the production of new antibodies to arise. ELISA for serum IgA revealed a significant increase in IgA levels after 3 wk of colonization (Fig. 1 A). IgA-secreting PC constitute a large fraction of LP –resident immune cells in conventional mice. We next decided to determine whether colonization with *T.mu* would increase the quantities of IgA-secreting cells in the intestinal LP. Immunofluorescence imaging of IgA, followed by quantification of IgA^+^ cells, indicated a significant increase in IgA-producing cells within the LP of *T.mu* colonized mice (Fig. 1 B). Transcytosis of IgA into the gut lumen is critically dependent on the expression of the *Pigr* on intestinal epithelial cells (IEC) (Johansen and Kaetzel, 2011). As engraftment of *T.mu* into the intestinal microbiota increased serum IgA levels and IgA^+^ cells in the LP, we hypothesized that luminal IgA levels and the expression of the IgA export machinery would be elevated in *T.mu* colonized mice. In support of this hypothesis, luminal IgA levels and the expression of *Pigr* showed a significant upregulation following colonization by *T.mu* (Fig. 1, C and D). These findings demonstrate that stable engraftment of *T.mu* into the intestinal tract promotes IgA production and transport across the intestinal epithelium. PC express the surface marker CD138 and are the major source of intestinal IgA (Sanderson et al., 1989; Pabst, 2012). To quantify absolute numbers of IgA^+^ PC, LP leukocytes were isolated and IgA and CD138 co-expressing cells were quantified. The LP of *T.mu* colonized mice displayed a striking elevation in IgA^+^CD138^+^ PC (identified as live, single cells, CD45^+^ CD11b^−^ B220^−^ MHCII^−/low^ CD138^+^ IgA^+^) compared with *T.mu*-free mice (Fig. 1 E). As IgA-secreting cells can be generated through Td and Ti pathways in gut-draining lymph nodes, we determined GC B cell and Tfh cell numbers in the MLN of control and *T.mu* colonized mice. In line with elevated IgA PC numbers, the MLN of *T.mu* colonized mice showed higher proportions of FAS^+^GL-7^+^ GC B cells and PD-1^+^CXCR5^+^ Tfh cells indicating a Td contribution to the elevated IgA levels (Fig. 1, F and G). The increase in PC, GC B cells, and Tfh cell frequencies was also reflected by higher absolute numbers of these cells (Fig. 1, E–G). *T.mu* preferentially colonizes the caecum of mice; however, Tfh cell frequencies were also elevated in the PP of the small intestine (Fig. 1 H). In line with this data, small intestinal IgA^+^ PC, similar to their large intestinal counterpart, were elevated in mice inoculated with gut commensal protist (Fig. 1 I). Elevated MLN Tfh cells were detectable 1 year after colonization by *T.mu* indicating a persistent mucosal immune modulation (Fig. 1 J). While IgA levels increased post-*T.mu* colonization, effects on the IgG isotype subclass were virtually absent (Fig. 1 K). Regulatory Foxp3^+^ T cells (Treg) have previously been associated with elevated IgA levels in mice; however, despite higher IgA levels, colonization by *T.mu* failed to result in a significant increase in Tregs numbers in the LP or MLN (Fig. S1 A). The observation of unchanged numbers of Tregs was further supported by steady TFG-β levels in the intestinal tract, a factor known to promote Treg differentiation and IgA CSR (Fig. S1 B) (Cazac and Roes, 2000; Wan and Flavell, 2007). TNFSF13B and TNFSF13, two TNF superfamily members previously reported to promote Ti CSR to IgA, did not change in expression in the presence of *T.mu* (Fig. S1 C). However, elevated levels of *Nos2* expression within the gut suggested a possible involvement of Ti CSR in the generation of IgA following colonization by *T.mu* (Fig. S1 C) (Tezuka et al., 2007). We previously reported an increase in TNF production in *T.mu*-colonized mice, further supporting the idea of Ti CSR as a mediator of the elevated IgA response (Chudnovskiy et al., 2016; Chiaranunt et al., 2022). Collectively, these results suggest both Td and Ti contribute to the increase in IgA observed in mice colonized with *T.mu*.

### *T.mu*-driven IgA is ICOS and T cell-dependent

**Figure 2.**
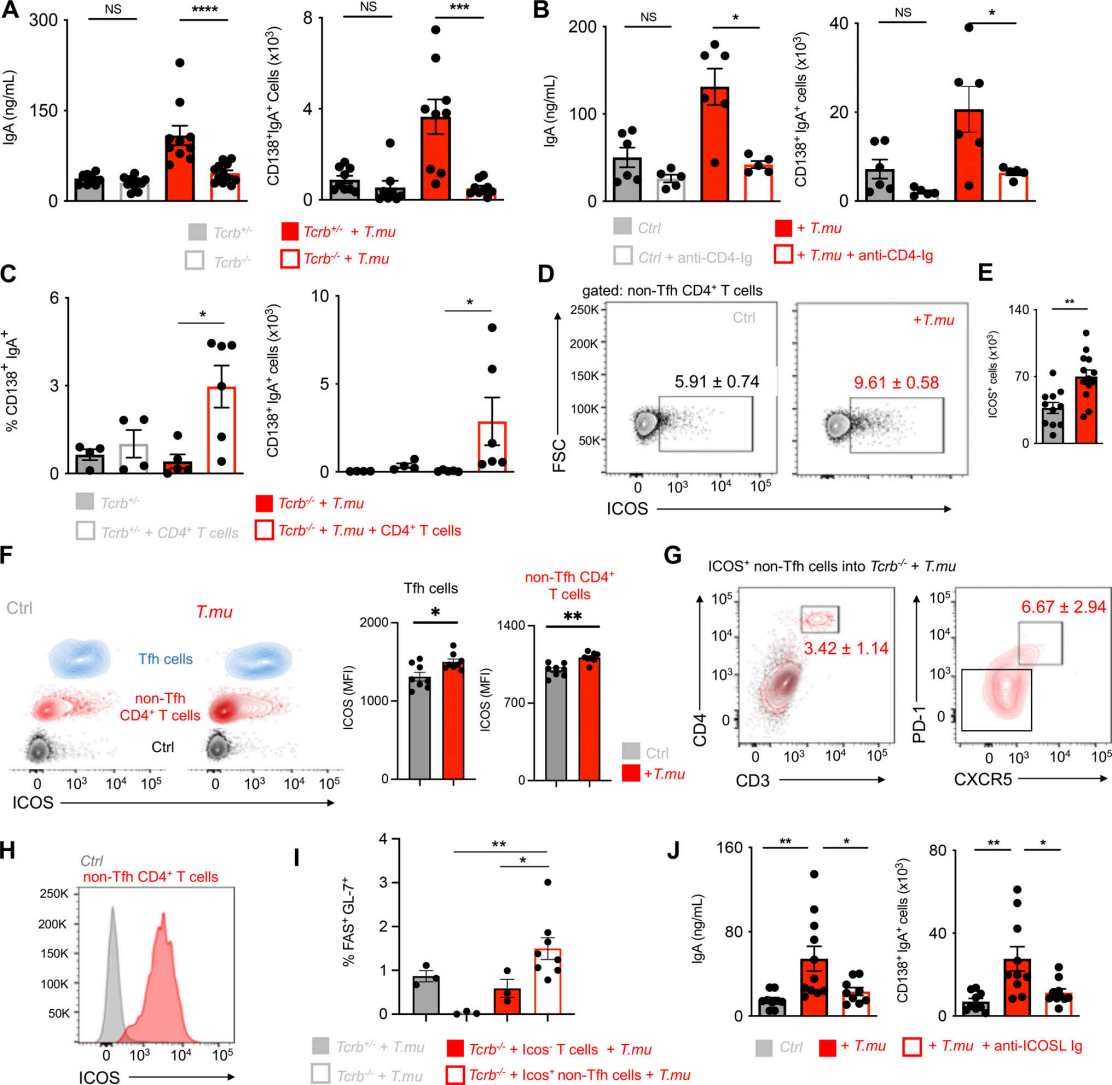
***T.mu* induces IgA in an ICOS and T cell-dependent fashion. (A)** Serum IgA levels and colonic lamina propria IgA^+^CD138^+^ plasma cell numbers in control or *T.mu*-colonized *Tcrb*^*+/−*^ and *Tcrb*^*−/−*^ mice. **(B)** Serum IgA levels and colonic lamina propria IgA^+^CD138^+^ plasma cell numbers in control or *T.mu* colonized with and without anti-CD4-Ig treatment. **(C)** Percentages and absolute number of colonic lamina propria IgA^+^CD138^+^ PC in control or *T.mu* colonized *Tcrb*^*+/−*^ and *Tcrb*^*−/−*^ after injection of CD4^+^ T cells. **(D)** Contour plots show ICOS expression on non-Tfh cells in MLN from control mice or mice colonized with *T.mu*. **(E)** Adjacent bar graph shows quantification of absolute ICOS^+^ CD4^+^ non-Tfh cells. **F)** ICOS expression on PD-1^+^CXCR5^+^ Tfh cells (blue), CD4^+^ non-Tfh cells (red), and isotype control staining on T cells (black) from control or *T.mu* colonized mice. Adjacent bar graphs show quantification of ICOS mean fluorescent intensity on PD-1^+^CXCR5^+^ Tfh cells and CD4^+^ non-Tfh cells. **(G)** Adoptive transfer of purified ICOS^+^ PD-1^*−*^ CXCR5^*−*^ CD4^+^ non-Tfh cells into *T.mu* colonized *Tcrb*^*−*/*−*^ mice. Contour plots show representative staining for CD3 and CD4 in *Tcrb*^*−/−*^ mice injected with PBS (black) or purified ICOS^+^ PD-1^*−*^ CXCR5^*−*^ CD4^+^ non-Tfh cells (red). CD4^+^ T cells in the MLN of mice injected with ICOS^+^ PD-1^*−*^ CXCR5^*−*^ CD4^+^ non-Tfh cells were analyzed for the expression of PD-1 and CXCR5. Numbers within plots show percentages ± SEM. **(H)** ICOS expression on ICOS^+^ PD-1^*−*^ CXCR5^*−*^ CD4^+^ non-Tfh cells after adoptive transfer into *T.mu* colonized *Tcrb*^*−/−*^ mice. **(I)** Percentages of FAS^+^GL-7^+^ GC B cells in the MLN of *T.mu* colonized *Tcrb*^*+/−*^, *Tcrb*^*−/−*^, or *Tcrb*^*−/−*^ mice injected with ICOS^*−*^ PD-1^*−*^ CXCR5^*−*^ CD4^+^ T cells or ICOS^+^ PD-1^*−*^ CXCR5^*−*^ CD4^+^ T cells. **(J)** Quantification of serum IgA levels and colonic IgA^+^CD138^+^ PC in control mice, mice colonized with *T.mu,* and *T.mu* colonized mice injected with blocking anti-ICOSL-Ig. Data shown are from two to three independent experiments with three to four mice/group. Mann–Whitney *U* test was performed. * = P ≤ 0.05, ** = P < 0.01, *** = P < 0.001, **** = P < 0.0001, NS = not significant. If not stated otherwise, displayed values include ± SEM.

To dissect the contribution of Td and Ti IgA CSR to the increase in IgA following colonization by *T.mu*, we first colonized *Nos2*^*−/−*^ or *Tnfa*^*−/−*^ mice and their respective age-matched, heterozygous littermate controls with *T.mu* to assess their LP IgA PC numbers. Both *Nos2* and *Tnfa* were dispensable for the *T.mu*-driven induction of IgA as both *Nos2*^*−/−*^ or *Tnfa*^*−/−*^ and their heterozygous littermate controls showed an equal induction of IgA after *T.mu* engraftment into the microbiota (Fig. S1, D and E). However, the redundancy for these factors does not formally exclude compensatory actions of TNF and iNOS in Ti-CSR following *T.mu* colonization. These findings prompted us to investigate whether genetic abrogation of T cell development or antibody-mediated depletion of CD4^+^ T cells would prevent the increased IgA levels following colonization with *T.mu*. To address this, groups of C57BL/6 and *Tcrb*^*−/−*^ were either left untreated or colonized with *T.mu* for 3 wk. Additional groups of C57BL/6 mice received control Ig or depleting anti-CD4 Ig injections for the course of 3 wk starting 3 days prior to *T.mu* colonization, leading to a full depletion of CD4^+^ T cells (Fig. S1 F). Measuring serum and fecal IgA levels in T cell–deficient or T cell–depleted mice failed to increase IgA levels, specifically in groups colonized with *T.mu* (Fig. 2, A and B). Notably, the absence of both T cells or B cells had a negative impact on the colonization efficiency by *T.mu* (Fig. S1 G). To causally demonstrate the requirement of CD4^+^ T helper cells in the *T.mu*-driven IgA induction, naïve, splenic, FACS-purified CD4^+^ T cells from wild type mice were injected i.v. into *Tcrb*^*−/−*^ control mice, or *Tcrb*^*−/−*^ mice colonized with *T.mu*. Analysis of IgA^+^ PC frequencies and numbers demonstrated the requirement of CD4^+^ T cells and Td IgA CSR in promoting IgA production following colonization with *T.mu* (Fig. 2 C).

We next determined if the increase in GALT-resident Tfh cells may be accompanied by changes in the expression of IgA CSR-promoting factors. To test this, we sorted Tfh cells from control mice and *T.mu* colonized mice and determined CD40L expression (Fig. S1 H), *Tbx21* and *Gata3* expression (Fig. S1 I), or *Il4*, *Il6*, *Il13*, *Il21*, *Il10* expression (Fig. S1 J). Surprisingly, no differences were observed, indicating an unaltered Tfh cell phenotype in the presence of *T.mu*. CSR has been reported to also occur in extrafollicular regions of the lymph nodes and spleen, where ICOS-expressing non-Tfh CD4^+^ T cells are suspected to promote this process (Muramatsu et al., 2000; Odegard et al., 2008). Supporting the idea that ICOS-expressing cells promote IgA, a recent report demonstrated that ICOSL deficiency minimizes IgA levels and antimicrobial IgA recognition (Landuyt et al., 2021). We therefore assessed ICOS expression on non-Tfh CD4^+^ T cells and observed a relative and quantitative increase in ICOS-expressing CD4^+^ non-Tfh cells in the MLNs of *T.mu*-colonized animals, supporting the hypothesis that *T.mu*-driven IgA requires ICOS:ICOSL interactions. (Fig. 2, D and E). Tfh cells and non-Tfh CD4^+^ T cells both expressed higher ICOS levels compared with their counterparts in *T.mu*-free animals (Fig. 2 F). ICOS expression on CD4^+^ T cells has been shown to promote Tfh cell differentiation upon ICOSL-driven stimulation, suggesting ICOS^+^ non-Tfh CD4^+^ T cells in supporting CSR (McAdam et al., 2001). To assess the potential of ICOS^+^ non-Tfh CD4^+^ T cells differentiation into Tfh cells, we adoptively transferred purified ICOS^+^ non-Tfh cells from the MLNs into *Tcrb*^*−/−*^, *T.mu*-colonized mice. Transferred ICOS^+^ non-Tfh cells differentiated into Tfh cells and retained high levels of ICOS, demonstrating their relationship to Tfh cells (Fig. 2, G and H). Investigating the ability of ICOS^+^ non-Tfh cells to promote GC B cell differentiation, we assessed GC B cell frequencies in the MLNs of *T.*mu colonized *Tcrb*^*+/−*^ mice, *Tcrb*^*−/−*^ mice, *Tcrb*^*−/−*^ mice injected with ICOS^*−*^ CD4^+^ T cells and *Tcrb*^*−/−*^ mice injected with ICOS^+^ non-Tfh cells. Adoptively transferred ICOS^+^ non-Tfh cells potently induced GC B cells at levels comparable with *Tcrb*^*+/−*^ mice (Fig. 2 I). To investigate the importance of ICOS on the induction of IgA following colonization by *T.mu*, we abrogated ICOS:ICOSL interactions through injections of blocking anti-ICOSL antibodies. Strikingly, this treatment almost completely reversed the elevated serum IgA levels and LP IgA PC numbers in mice colonized with *T.mu* (Fig. 2 J).

**Figure S2.**
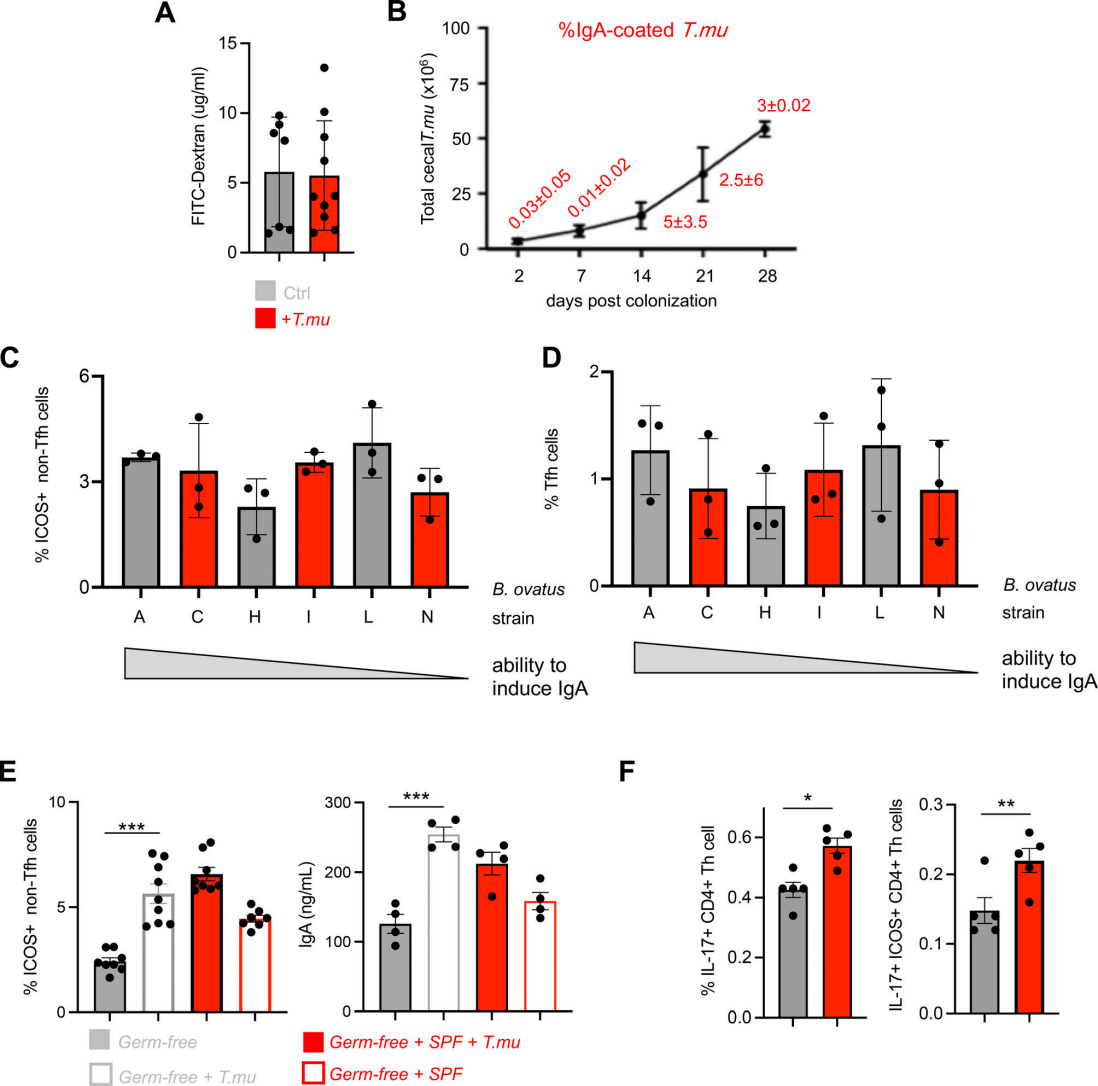
***T.mu* promotes ICOS**^**+**^ **CD4**^**+**^ **non-Tfh cells independent of the microbiota. (A)** Concentrations of FITC-Dextran in the serum of control or *T.mu* colonized mice. **(B)** Total *T.mu* numbers in the caecum of *T.mu* colonized C57BL/6 mice, 2, 7, 14, 21, and 28 days after colonization. Staining of *T.mu* with anti-IgA was performed at the indicated time points. Red numbers adjacent to the curve indicate percentages of IgA-coated *T.mu* ± SEM. **(C and D)** Groups of germ-free mice were colonized with *B*.* ovatus* strains (A, H, N, I, C, and L). Three weeks after colonization ICOS^+^ CD4^+^ non-Tfh cells and PD-1^+^ CXCR5^+^ Tfh cells were quantified in the MLN. **(E)** Groups of germ-free mice were either left untreated, or were colonized with purified *T.mu*, purified *T*.*mu,* and a complete SPF microbiota, or a complete SPF microbiota only. ICOS^+^ CD4^+^ non-Tfh cells were quantified in the MLN and serum IgA levels were assessed 3 wk after colonization. **(F)** Frequencies of IL-17 producing Th cells and ICOS^+^ CD4^+^ non-Tfh cells were assessed in the MLN of control of *T*.*mu*-colonized mice. Data shown are representative of at least two to three independent experiments with three to four mice/group. If not stated otherwise, displayed values include ± SEM.

To evaluate whether induction of IgA is a consequence of an intestinal barrier breach, we compared intestinal barrier integrity using oral application of FITC–dextran into control or *T.mu*^+^ mice as previously reported, but failed to observe any notable difference between the experimental groups (Fig. S2 A) (Bhinder et al., 2014). We next investigated whether *T.mu* could be the target of IgA antibodies and assessed the IgA coating of *T.mu* over the course of intestinal colonization. While intestinal *T.mu* counts followed an anticipated increase, IgA binding to *T.mu* was only observed in up to 5% of the intestinal protozoa (Fig. S2 B). To determine whether ICOS expression on non-Tfh CD4^+^ T cells is a consequence of microbial colonization, we analyzed the CD4^+^ T cell compartment of germ-free mice following colonization with previously reported IgA-inducing commensal bacteria. We colonized germ-free mice with six distinct *Bacteroides ovatus* strains (A, C, H, I, L, N) that covered a graded capability to promote IgA CSR (A > C > H > I > L > N) (Yang et al., 2020). 3 wk after colonization, MLN PD-1 ^+^CXCR5^+^ Tfh cells and ICOS^+^ non-Tfh cells were quantified. *B*. *ovatus* strains previously reported to induce high levels of IgA failed to differentially increase Tfh cells and ICOS-expressing Tfh cells in relation to their previously reported IgA-inducing capacities (Fig. S2, C and D). In contrast, monocolonization of germ-free mice with quadruple-sorted *T.mu* demonstrated that protozoan colonization was sufficient to promote an increase in ICOS^+^ non-Tfh CD4^+^ T cells and serum IgA (Fig. S2 E). We previously reported an increase in IL-17–producing CD4^+^ T cells in the colonic LP of mice colonized with *T.mu* (Chudnovskiy et al., 2016). Comparing IL-17 levels in CD4^+^ T cells and ICOS^+^ non-Tfh cells in the MLNs of *T.mu*^+^ animals revealed a slight, yet significant increase in IL-17 production by these cells (Fig. S2 F). While IL-17 is dispensable for the differentiation of Tfh cells and the induction of IgA, IL-17R signaling has been reported to promote *Pigr* expression, a feature observed following *T.mu* colonization (Fig. 1 D) (Cao et al., 2012). *T.mu* colonization thus imposes a mode of immune regulation that promotes IgA. In conclusion, these results demonstrate that *T.mu*-mediated IgA induction operates through ICOS-expressing Tfh and ICOS^+^ non-Tfh cells. A reduced antibacterial IgA reactivity, as a consequence of a defective ICOS:ICOSL interaction, has previously been reported, suggesting that colonization by the protozoan commensal *T.mu* may improve anti-bacterial IgA recognition through the upregulation of ICOS on CD4^+^ T cells and the increase in Tfh cells (Landuyt et al., 2021).

### Shift in microbial composition and IgA-reactome following colonization with *T.mu*

**Figure 3.**
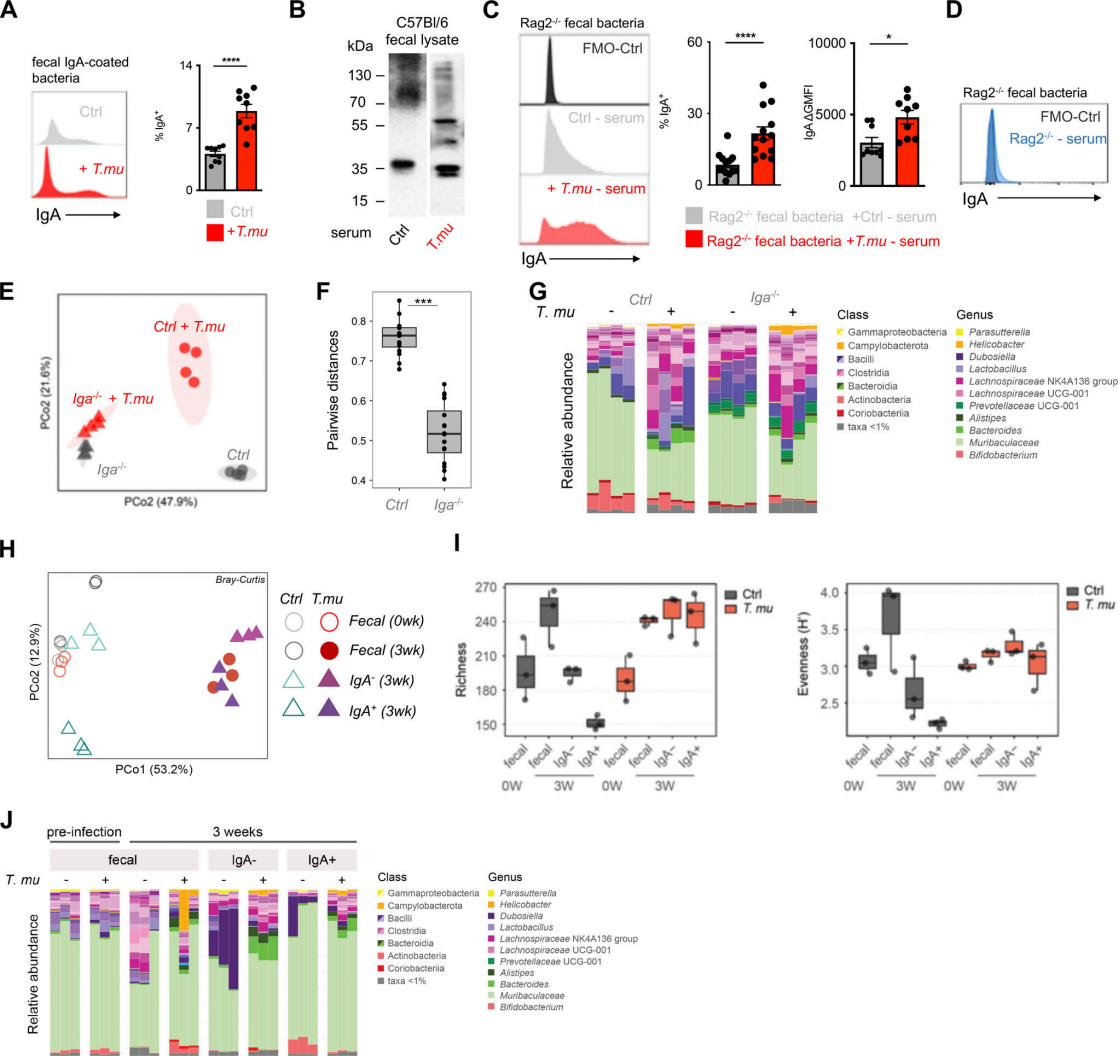
**T*.mu* shapes the anti-bacterial IgA reactome. (A)** Fecal bacteria were isolated from control or *T.mu* colonized mice and stained with anti-IgA antibodies. Histograms show staining of anti-IgA antibodies. Adjacent bar graph shows the quantification of IgA-coated bacteria. **(B)** Western blot analysis of IgA reactivity on wild type C57BL/6 fecal bacterial protein lysates. Membranes were developed after incubation with serum from control mice or *T.mu* colonized mice. Numbers adjacent to membranes indicate molecular weight. **(C)** Serum from control mice or *T.mu* colonized mice was collected and used to stain fecal bacteria obtained from *Rag2*^*−/−*^ mice. Histogram shows coating of fecal bacteria with serum IgA. Adjacent bar graphs show quantification of IgA-coated bacteria and the serum staining intensity displayed as mean fluorescent intensity normalized to controls. **(D)** Fecal bacteria obtained from *Rag2*^*−/−*^ mice were stained with serum from *Rag2*^*−/−*^ mice (blue histogram). **(E)** PCoA of 16S rDNA gene sequencing data obtained from fecal samples collected from control and *T.mu* colonized littermates and *Iga*^*−/−*^ mice. **(F)** Pairwise distance comparison of microbial communities in littermate controls and *Iga*^*−/−*^ mice free of or colonized with *T.mu*. **(G)** Relative abundance of bacterial OTUs in littermate control and *Iga*^*−/−*^ mice free of or colonized with *T.mu*. colored by phylum as indicated. **(H)** PCoA of 16S rDNA gene sequencing data obtained from fecal samples or sorted IgA^*−*^ and IgA-coated bacteria, collected from control and *T.mu* colonized mice. **(I)** Bacterial richness of fecal and flow-sorted microbiota from control or *T.mu* colonized mice Bacterial evenness of fecal and flow-sorted microbiota from control or *T.mu* colonized mice. **(J)** Relative abundance of bacterial OTUs in fecal and flow sorted microbiota from control or *T.mu* colonized mice. Data shown is representative of at least one to three independent experiments with three to four mice/group. Mann–Whitney *U* test was performed. * = P ≤ 0.05, ** = P < 0.01, *** = P < 0.001, **** = P < 0.0001, NS = not significant. If not stated otherwise, displayed values include ± SEM. Source data are available for this figure: SourceData F3.

**Figure S3.**
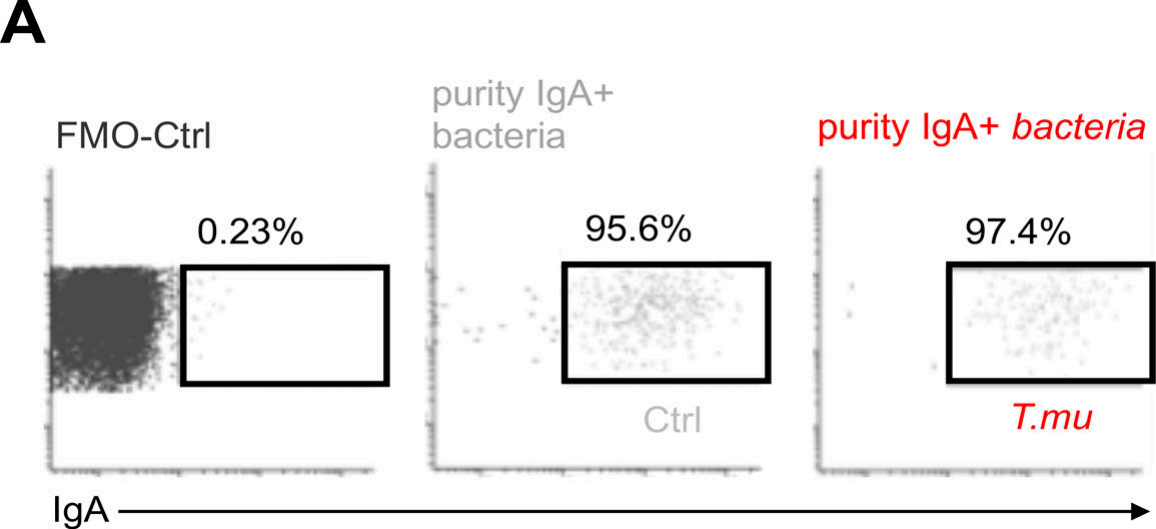
**Purity of sorted IgA-coated bacteria. (A)** Post sort analysis of IgA-coated bacteria purified from control or *T*.*mu* colonized mice. Values adjacent to gates indicate percentages.

To test the hypothesis that colonization with *T.mu* changes the antibacterial IgA reactivity, fecal bacteria were isolated from control or *T.mu* colonized mice and stained with anti-IgA antibodies to determine the degree of IgA coating. Mice colonized by *T.mu* displayed significantly higher IgA coating compared with control mice, supporting Landuyt et al., 2021’s data of an ICOS:ICOSL-dependent regulation of antimicrobial IgA (Fig. 3 A) (Landuyt et al., 2021). To examine whether anti-commensal IgA antibodies show a distinct reactivity toward luminal bacteria in the presence of *T.mu*, equal concentrations of fecal protein from bacterial lysates of conventional C57BL/6 mice were separated by SDS-PAGE and transferred onto nitrocellulose membranes. Membranes were separated and incubated with control serum or serum collected from *T.mu* colonized mice. Anti-IgA binding to the blotted bacterial proteins was detected using anti-mouse IgA secondary antibodies. Membranes developed under identical conditions revealed that IgA antibodies from *T.mu* colonized mice exhibited a distinct band pattern when compared with IgA antibodies from control mice, showing that serum IgA antibodies from *T.mu* colonized mice have a distinct IgA reactivity against bacterial proteins (Fig. 3 B). Sera obtained from control mice and mice colonized with *T.mu* recapitulated the elevated IgA-coating pattern even when fecal bacteria from antibody-deficient *Rag2*^*−/−*^ mice were used as a template for IgA coating (Fig. 3 C). As expected, serum from *Rag2*^*−/−*^ failed to induce IgA coating on the microbiota obtained from *Rag2*^*−/−*^ mice (Fig. 3 D). These findings demonstrate that serum IgA and secreted IgA display an elevated capacity to bind bacteria after colonization with *T.mu*. We recently demonstrated that colonization by *T.mu* alters the timely adaptation of the gut microbiota after colonization in a B cell-dependent fashion (Popovic et al., 2024). This study investigated microbial composition in *µMT*^*−/−*^ mice that have a reported defect in B cell development and antibody production, particularly IgM and IgG, while retaining the ability to generate IgA (Macpherson et al., 2001). We therefore asked whether induction of IgA following *T.mu* colonization could impact the shift in microbial composition (Popovic et al., 2024). Control or *Iga*^*−/−*^ mice were colonized with *T.mu* and their bacterial communities were compared using 16S rDNA sequencing. Principal coordinate analysis, based on Bray–Curtis compositional dissimilarities, revealed distinct clustering between microbial communities in control and *Iga*^*−/−*^ mice in the presence of *T.mu* (Fig. 3 E). Pairwise distances were significantly increased in IgA-sufficient control mice when compared with *Iga*^*−/−*^ mice, indicating greater differences in bacterial communities following *T.mu* colonization in the presence of IgA (Fig. 3 F). Analyzing communities at the genus level mirrored the IgA-dependent shift in the microbial communities, suggesting that colonization by *T.mu* shapes the bacterial composition in an IgA-dependent manner (Fig. 3 G). These results inspired us to purify fecal IgA-coated and non-coated bacteria from control and *T.mu* colonized mice to determine their composition. DNA was isolated from FACS-purified IgA-coated bacteria that were sorted at 95.6 or 97.4% purity for control and *T.mu*-colonized mice, respectively (Fig. S3 A). 16S rDNA gene sequencing and principal coordinate analysis, based on Bray–Curtis compositional dissimilarities, demonstrated a separation of IgA-coated and non-coated bacteria in control and *T.mu* colonized mice. Bacterial composition obtained from mice colonized with *T.mu* clustered distinctly from control samples, supporting a change in the profile of IgA-reactive bacteria in the presence of the protist (Fig. 3 H). At week 3, although fecal bacterial richness was similar in naïve and *T.mu* colonized mice, both uncoated and IgA-coated bacteria showed greater dissimilarity in *T.mu* colonized mice, suggesting an increase in IgA reactivity in the presence of *T.mu* (Fig. 3 I). Evenness across microbial communities displayed similar trends, albeit with elevated fecal diversity in control mice. While the bacterial evenness remained consistently distributed, increased richness of OTUs was observed in mice colonized by *T.mu* (Fig. 3 I). The recorded elevation in microbial richness in *T.*mu colonized mice was also reflected when comparing the relative abundance of taxa across the different experimental groups, further emphasizing the broadening in the reactivity of IgA in the presence of *T.mu* (Fig. 3 J). Collectively, these findings demonstrate that colonization with the protozoan commensal *T.mu* imposes an IgA-dependent change on the gut microbiota that is accompanied by an increase in the IgA reactivity toward intestinal bacteria.

### B cell–expressed MHCII is essential for *T.mu*-driven IgA

Bacterial antigens are translocated through epithelial microfold cells and subsequently transferred to MHCII-expressing DC to facilitate the transport of the antigen to the T cell zone of the draining lymph node (Brandtzaeg, 2009; Neutra et al., 1996). ICOS expression on T cells and the expansion of Tfh cells have been demonstrated to require T cell receptor (TCR) engagement through MHCII. As *T.mu* colonization elevates the numbers of ICOS^+^ CD4^+^ non-Tfh cells and Tfh cells, we reasoned that *T.mu*-mediated induction of IgA and ICOS may also depend on MHCII-mediated antigen presentation (Ise et al., 2018; Deenick et al., 2010).

**Figure 4.**
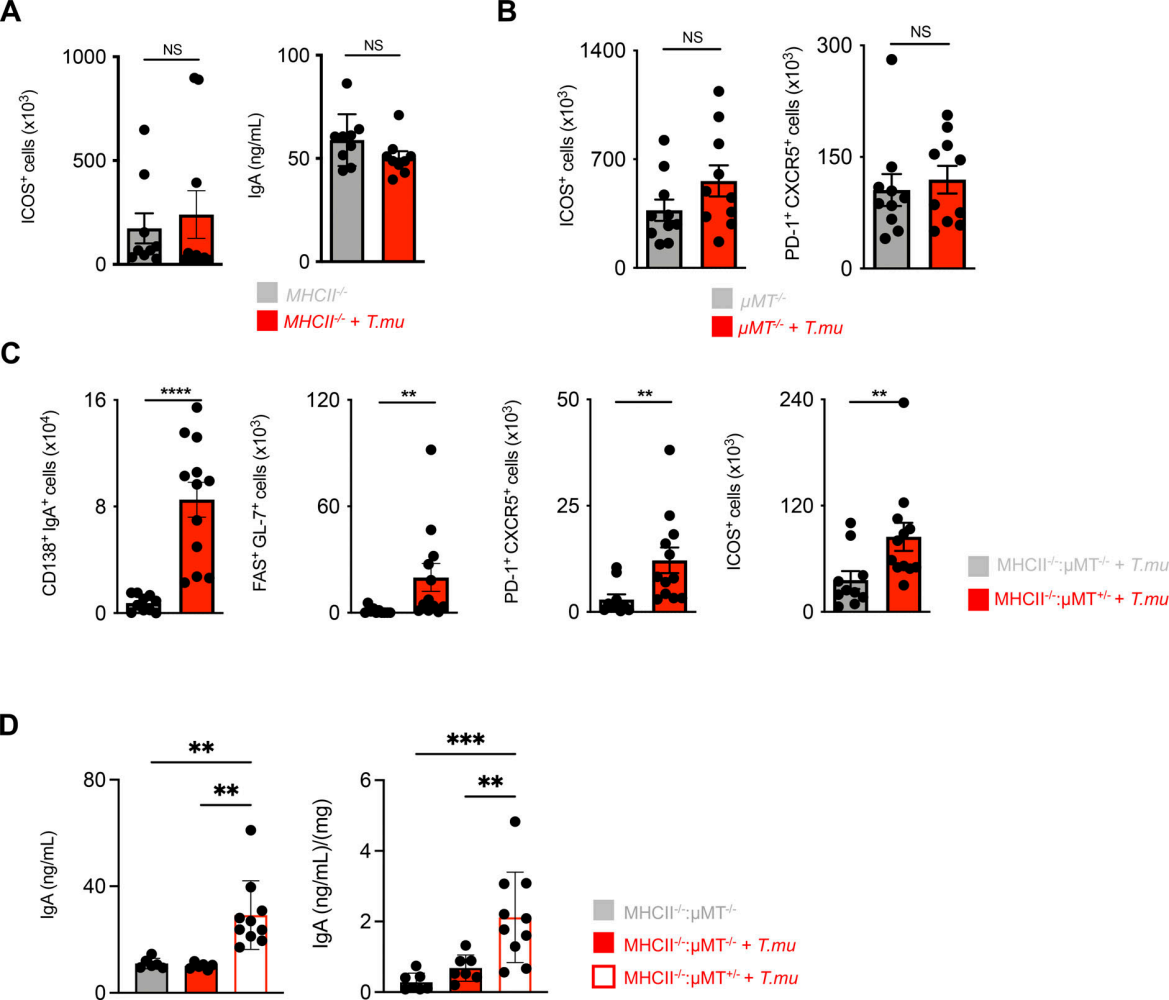
***T.mu*-driven IgA requires antigen-presentation by B cells. (A)** Quantification of ICOS^+^ CD4^+^ non-Tfh cells in the MLN and serum IgA levels in control and *T.mu* colonized *MHCII*^*−/−*^ mice. **(B)** Quantification of MLN ICOS^+^ CD4^+^ non-Tfh cell and Tfh cell numbers in *µMT*^*−/−*^ control mice or *µMT*^*−/−*^ mice colonized with *T.mu*. **(C)** Analysis of bone marrow chimeric mice. Groups of mice received a 50:50 mixture of either *MHCII*^*−/−*^ *µMT*^*−/−*^ or *MHCII*^*+/−*^ *µMT*^*+/−*^ bone marrow cells after lethal irradiation and were colonized with *T.mu* after full bone marrow reconstitution. Absolute numbers of colonic IgA^+^CD138^+^ PC, MLN FAS^+^GL-7^+^ GC B cells, PD-1^+^CXCR5^+^ Tfh cells, and ICOS^+^ CD4^+^ non-Tfh cells were quantified. **(D)** Serum and fecal IgA levels in control or *T.mu* colonized *MHCII*^*−/−*^ *µMT*^*−/−*^ mice and *T.mu*-colonized *MHCII*^*−/−*^ *µMT*^*+/−*^ mice. Data shown are representative of at least three independent experiments with three to four mice/group. Mann–Whitney *U* test was performed. * = P ≤ 0.05, ** = P < 0.01, *** = P < 0.001, **** = P < 0.0001, NS = not significant. If not stated otherwise, displayed values include ± SEM.

To address this question, we left *MHCII*^*−/−*^ mice untreated or colonized them with *T.mu* to determine ICOS^+^ CD4^+^ non-Tfh cells, Tfh cell numbers, and IgA levels. In contrast to age-matched littermate control mice, *MHCII*^*−/−*^ mice failed to increase in ICOS^+^ CD4^+^ non-Tfh cells, serum IgA, and Tfh cells in the presence of *T.mu* (Fig. 4 A). These findings indicate that protozoa-driven IgA induction and T cell expansion require MHCII–TCR interactions. Naive B cells are underappreciated antigen-presenting cells that have recently gained attention as a critical source of MHCII that promotes the anti-plasmodium antibody response (Thouvenel et al., 2021). We hypothesized that B cell-presented antigens are mandatory for the ICOS-dependent, anti-commensal IgA response driven by colonization with *T.mu*. To test this hypothesis, we colonized B cell-deficient *µMT*^*−/−*^ mice with *T.mu* and determined the numbers of ICOS^+^ CD4^+^ non-Tfh cells and Tfh cells in the draining lymph nodes. In support of our hypothesis, we observed that *µMT*^*−/−*^ mice failed to show a significant increase in ICOS^+^ CD4^+^ non-Tfh cells (Fig. 4 B). Tfh cells further required B cells for their expansion and consequently failed to increase in *µMT*^*−/−*^ mice after protozoan colonization, demonstrating that B cells are a crucial element in supporting the *T.mu*-driven expansion of MHCII-dependent, ICOS^+^ CD4^+^ non-Tfh cells, and Tfh cells (Fig. 4 B). To determine whether MHCII on other hematopoietic cells (e.g., macrophages or DC) could contribute to the anti-commensal IgA response after colonization with *T.mu*, bone marrow chimeric mice were generated. A group of lethally irradiated *µMT*^*−/−*^ mice received a 50:50 mixture of *MHCII*^*−/−*^:*µMT*^*−/−*^ bone marrow cells while the control group of *µMT*^*−/−*^ mice was transplanted with a 50:50 mixture of *MHCII*^*+/−*^:*µMT* ^*±*^ bone marrow cells. We hypothesized that a reconstituted *MHCII*^*−/−*^:*µMT*^*−/−*^ immune system, comprised of only MHCII-deficient B cells, would fail to show an increase in the *T.mu*-driven IgA response and expansion of ICOS^+^ CD4^+^ non-Tfh and Tfh cells. Following full bone marrow reconstitution, mice were orally colonized with *T.mu* and analyzed for mucosal IgA-producing PC, GC B cells, Tfh cells, and ICOS^+^ CD4^+^ non-Tfh cells. In line with our hypothesis, and supported by the observations in *µMT*^*−/−*^ and *MHCII*^*−/−*^ mice, colonization by *T.mu* only yielded a full IgA response in mice capable of presenting antigen via B cell-specific MHCII (Fig. 4 C). In addition, analysis of serum and fecal IgA levels revealed that *T.mu* colonization exclusively increased IgA in the presence of MHCII-sufficient B cells (Fig. 4 D). These findings demonstrated that protozoan colonization initiates an ICOS- and T cell–dependent IgA response through MHCII antigen presentation by B cells. Colonization with *T.mu* thus elevates GC B cells, ICOS^+^ CD4^+^ non-Tfh cells, Tfh cells, and IgA PC to promote new IgA reactivity against luminal bacterial antigens. These observations inspired us to ask whether the underlying pathway of immune activation following colonization by *T.mu* could be harnessed to improve the IgA response against non-bacterial antigens in the form of a natural adjuvant.

### Colonization with *T.mu* promotes peripheral dissemination of gut-primed PC

**Figure 5.**
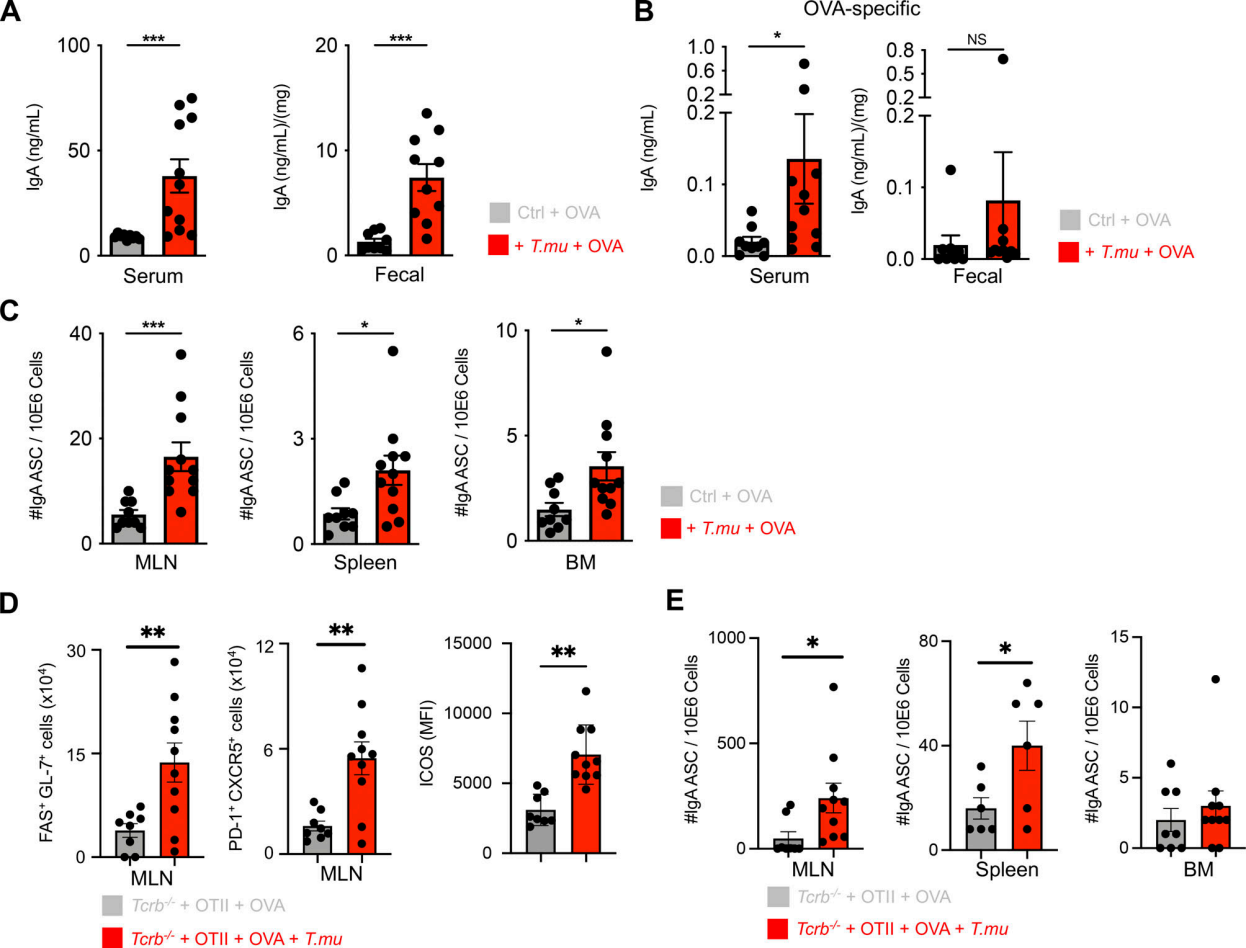
***T.mu* promotes peripheral dissemination of IgA-secreting plasma cells primed against gut antigens. (A)** Groups of control or *T.mu* colonized mice were treated with OVA-containing drinking water ad libitum for 3 wk. Total serum and fecal IgA levels were assessed. **(B)** OVA-specific IgA levels were determined in the serum or feces of OVA-treated mice in the presence or absence of *T.mu*. **(C)** MLN, spleens, and bone marrow were harvested from mice used in A, and numbers of OVA-specific anti-IgA–secreting cells (ASC) were determined. **(D)** Purified CD4^+^ *OTII*^*Tg*^ T cells were adoptively transferred into control or *T.mu* colonized *Tcrb*^*−/−*^ mice followed by treatment with OVA-containing drinking water. MLNs were analyzed for FAS^+^GL-7^+^ GC B cells, PD-1^+^CXCR5^+^ Tfh cells, and ICOS expression on non-Tfh cells. **(E)** MLN, spleens, and bone marrow were harvested from animals used in D, and numbers of OVA-specific anti-IgA secreting cells (ASC) determined. Data shown are representative of at least two to three independent experiments with three to four mice/group. Mann–Whitney *U* test was performed. * = P ≤ 0.05, ** = P < 0.01, *** = P < 0.001, **** = P < 0.0001, NS = not significant. If not stated otherwise, displayed values include ± SEM.

PC, primed against gut luminal antigens, have been reported to egress from the LP and migrate to peripheral tissues (Mei et al., 2009; Rojas et al., 2019). We reasoned that the increased anti-commensal IgA response driven by *T.mu* would also improve an IgA response against non-bacterial orally ingested antigens and mediate the peripheral dissemination of antigen-specific PC. To test our hypothesis, groups of mice were either left untreated or colonized with *T.mu* followed by oral exposure to the model antigen Ovalbumin (OVA). OVA-containing drinking water was provided ad libitum for 3 consecutive weeks followed by the analysis of OVA-specific serum IgA levels and OVA-specific IgA-secreting PC in peripheral organs. Oral exposure to OVA commonly elicits very weak antigen-specific IgA titers in conventional wild type mice, indicating that in the absence of protozoa, the microbiota has a weak adjuvant activity toward IgA (Israf et al., 2004). In accordance with our previous observations, colonization by *T.mu* increased serum and fecal IgA levels even in the presence of OVA (Fig. 5 A). In contrast to the microbiota’s weak adjuvanticity toward OVA, *T.mu* drives elevated levels of OVA-specific serum and fecal IgA antibodies in colonized mice when compared with controls (Fig. 5 B). This demonstrates that a *T.mu*-boosted mucosal IgA response potentiates IgA against non-microbial, luminal antigens (Fig. 5, A and B). To investigate if anti-OVA IgA-secreting PC would display improved peripheral dissemination, ELISPOT assays of OVA-specific IgA PC were performed on MLN, spleen, and bone marrow cells. In line with increased serum and fecal anti-OVA IgA response, *T.mu* colonization resulted in significantly higher peripheral dissemination of OVA-specific IgA-secreting PC (Fig. 5 C). To investigate whether *a T.mu*-promoted IgA response would also operate in the sole presence of OVA-specific T cells, we adoptively transferred CD4^+^ *OTII*^*Tg*^ *Rag2*^*−/−*^ T cells into either untreated or *T.mu* colonized *Tcrb*^*−/−*^*Tcrd*^*−/−*^ mice via i.v. injection. Following adoptive transfer, mice were provided with OVA-containing drinking water for 3 wk. *OTII*^*Tg*^ CD4^+^ T cells induced significantly elevated GC B cells, Tfh cell numbers, and elevated ICOS-expression on ICOS^+^ CD4^+^ non-Tfh cells in the presence of *T.mu* (Fig. 5 D). In line with this data, increased anti-OVA IgA-secreting PC were observed in the MLN and spleen (Fig. 5 E). These findings demonstrate that the commensal protozoa *T.mu* functions as natural driver for luminal antigens by boosting the Td induction of mucosal IgA and enhancing the peripheral dissemination of gut-primed antigen-specific PC.

## Discussion

Our study uncovers an underappreciated contribution by the recently identified protozoan commensal *T.mu* to the mucosal and systemic IgA. Although the impact of the gut microbiota on the induction of IgA is accepted, the impact of protozoan commensals within the microbiota has only been addressed by a limited number of studies, preferably focusing on pathogenic protozoa (Tan et al., 2021; Reperant et al., 1992). Here, we report that colonization with the commensal protozoa *T.mu* elicits substantial changes in the host’s IgA production, IgA reactivity, and PC dissemination. The increase in IgA and the changes in IgA reactivity are the result of newly generated IgA-producing PC that arise through a Td mechanism. This is due to an increased GC response and a population of ICOS^+^ CD4^+^ non-Tfh cells, which retain high levels of ICOS expression and can differentiate into Tfh cells. Moreover, ICOS:ICOSL co-stimulation and antigen-presentation by B cells appear to be uniquely required for this response to elevate serum and fecal IgA levels. The intestinal microbiota of *T.mu* colonized mice is distinctly shaped in an IgA-dependent fashion, coinciding with a greater coating of gut bacteria by IgA. IgA-coated bacteria show greater richness and evenness, indicating an association between broader IgA reactivity toward luminal commensal bacteria and *T.mu* colonization. Importantly, the elevated induction of IgA in mice carrying *T.mu* additionally promotes IgA reactivity to non-bacterial, orally ingested antigens, accompanied by increased peripheral dissemination of PC primed against luminal antigens. This response is independent of the host’s reactivity against the commensal protist, emphasizing effects by the protist that are similar to those of an adjuvant. These observations extend our understanding of how the microbiota influences the host’s adaptive immune response and highlights a non-bacterial commensal as an important regulator of the homeostatic mucosal IgA response.

The role of *Tritrichomonas* spp. as the driver of the host’s immune response has multiple effects on the intestinal tract. Colonization of mice with *T.mu* potentiates antimicrobial immunity against intracellular pathogens like *Salmonella* or *Clostridium* (Chiaranunt et al., 2022; Chudnovskiy et al., 2016). However, the elevated threshold of immunity imposed by *T.mu* confers risks to the host’s health and can exacerbate disease outcomes under permissive circumstances (Chudnovskiy et al., 2016; Escalante et al., 2016). In contrast to these findings are *T.mu*’s ability to promote the anti-inflammatory effects of gut-derived PC in the central nervous system (CNS), suggesting a divergent role of *T.mu* in regulating peripheral diseases (Rojas et al., 2019). While IgA is dispensable for ameliorated pathology in the CNS, peripheral dissemination of PC and the release of other PC-related factors arise as intriguing targets for future research (Rojas et al., 2019). Moreover, the expansion of ICOS-expressing CD4^+^ non-Tfh cells warrants attention and suggests that an increase in undifferentiated Tfh cells in the GALT of mice promotes Td IgA CSR in an ICOS-dependent fashion (Ma et al., 2012). New experimental approaches are required to selectively address these observations and dissect the distinct contribution and developmental trajectories of ICOS-expressing CD4^+^ non-Tfh cells. The observation that B cell-expressed MHCII contributes to the expansion of IgA PC and the GC response in the presence of *T.mu* is another exciting observation that implicates gut microbes as a driver of an increased IgA response through antigen presentation by B cells. While the role of B cells as inducers of IgA has been reported for a conserved epitope in the N-terminus of the circumsporozoite protein in *Plasmodium falciparum*, other protozoan or bacterial epitopes may also be the target of such response (Thouvenel et al., 2021). These observations challenge the role of conventional DC and FDC as sole antigen-presenting cells for mucosal IgA responses and demand future investigations to dissect the distinct roles of each antigen-presenting cell type in mucosal IgA production (Tezuka et al., 2007).

IgA responses against mucosal antigens are of growing interest for the development of vaccines against emerging mucosal pathogens (Boyaka, 2017). Considering that the microbiota impacts the systemic vaccine response, a stratification around a protozoan prebiotic formulation may be an attractive approach to tailor the outcome of vaccinations toward a sustained and improved IgA response. In line with this notion, supplementing the gut microbial community with a protozoan commensal like *T.mu* may serve as natural adjuvants for mucosal IgA and offer a promising approach to drive IgA responses to orally ingested antigens. Human relatives of *T.mu* have been reported in the intestines of healthy volunteers (Chudnovskiy et al., 2016). Whether colonization of humans by these commensal protozoa proves advantageous for the induction of mucosal IgA toward orally ingested antigens remains an unexplored area of research that could aid in explaining population heterogeneity and variations in mucosal IgA responses.

Collectively, we report multiple novel observations centered around the host’s IgA response in the presence of a previously underappreciated gut commensal microbe. The uncovered modes of action for a protozoan-boosted IgA response use a Td pathway that operates along the axis of ICOS:ICOSL co-stimulation and B-cell specific MHCII expression. This pathway additionally promotes IgA responses against non-microbial luminal antigens and may be of benefit to the host in tolerating new dietary antigens. Protozoan commensals like *T.mu* represent an interesting, yet underexplored group of microbes for future investigations into innovative natural adjuvant formulations that enhance the induction of IgA with beneficial effects for the induction of oral tolerance and host defense.

## Materials and methods

### Animals

C57BL/6J, B6;129S-*Tnf*^*tm1Gkl*^/J (*Tnfa*^*−/−*^), B6.129P2-*Nos2*^*tm1Lau*^/J (*Nos2*^*−/−*^), B6.129S2-*H2*^*dlAb1-Ea*^/J (*MHCII*^*−/−*^), and B6.129S2-*Ighm*^*tm1Cgn*^/J (*µMT*^*−/−*^) mice were purchased from the Jackson Laboratories. *Iga*^*−/−*^ mice were kindly provided by Dr. Dan Wiener (Buck Institute for Research on Aging, Novato, CA, USA) and Dr. Margaret E. Conner (Baylor College of Medicine, Houston, TX, USA). Spleens from B6.Cg-Tg(TcraTcrb)425Cbn/J (OTII^Tg^ Thy.1.1 background) were kindly provided by Dr. Tracy McGaha (Princess Margaret Cancer Center, University Health Network, Toronto, Canada). Germ-free animals were obtained through the Gnotobiotic facility at the University of Toronto Division of Comparative Medicine animal facility. Conventional mice were maintained in specific pathogen-free (SPF) conditions at the University of Toronto Division of Comparative Medicine animal facility. Mice were used at the age of 6–12 wk. Experiments were conducted using age- and sex-matched littermate controls. Animals were housed in a closed caging system and provided with an irradiated chow diet (Envigo Teklad 2918) and acidified water (reverse-osmosis and UV-sterilized) with a 12 h light/dark cycle. Following colonization with *Tritrichomonas musculis*, animals were housed in separate cages to avoid contamination with non-colonized mice. Animal experiments were approved by the Local Animal Care Committee (LACC) at the Faculty of Medicine, University of Toronto.

### Colonization of germ-free animals

Germ-free C57BL/6 mice were bred in isolators at the Microbiome Translational Center Gnotobiotic Facility at the Icahn School of Medicine. Mice 6–8 wk of age were colonized by oral gavage with one of six strains of *B*. *ovatus* (cultured anaerobically in rich BHI media as in Yang et al. [2020]) and then housed in barrier cages under aseptic conditions. Experiments were approved by the Institutional Animal Care and Use Committee at the Icahn School of Medicine at Mount Sinai. For the monocolonization of germ-free animals with *T.mu*, 2 × 10^6^ quadruple FACS-sorted *T.mu* were resuspended in sterile PBS and gavaged into germ-free recipients. Colonization of germ-free mice with SPF microbiota was performed as previously described (Chudnovskiy et al., 2016). Germ-free animals were conventionalized with a complete SPF microflora for 4 wk, prior to gavage with sorted *T.mu*. All animals were analyzed 3 wk after gavage with *T.mu*.

### *Tritrichomonas* colonization

Purification of *T.mu* was performed as described in Chudnovskiy et al. (2016). The caecal content of *T.mu* colonized mice was harvested into sterile PBS and filtered through a 70-µm cell strainer. Cecal contents were spun at 500 *g* for 7 min at 4°C. The supernatant was discarded and the pellet was washed twice with sterile PBS. Protozoa were enriched through a Percoll gradient and the interphase was collected, washed, and filtered through a 70-µm cell strainer. The *T.mu* were sorted into sterile PBS on a BD Influx using the 100-µm nozzle at 27 psi at 4°C at a purity of >99%. Sorted *T.mu* were spun down and resuspended in sterile PBS prior to orally gavaged 2 × 10^6^ cells into mice.

### Quantification of *T.mu* colonization

Quantification of *T.mu* was performed as previously described (Chudnovskiy et al., 2016). In brief, caeca were harvested and opened, and the caecal content was resuspended in 10 ml of sterile PBS. Protozoa were counted using a hemocytometer.

### Isolation of lymphocytes

LP lymphocytes were isolated as described in Burrows et al. (2020). The large intestines were incubated in Hanks’ balanced salt solution (HBSS w/o Ca^2+^Mg^2+^; GIBCO) with EDTA (5 mM) and HEPES (5 mM) for 10 min at 37°C with mild agitation. Samples were vortexed for 10 s and washed in 37°C HBSS buffer (HBSS with Ca^2+^Mg^2+^; GIBCO). Tissues were minced into ∼1 mm pieces and transferred into 10 ml of digestion buffer containing HBSS with Ca^2+^Mg^2+^, HEPES (5 mM), FBS (2%), collagenase, and DNaseI. Digested samples were vortexed for 10 s and filtered with a 70-µm cell strainer, and pellets containing leukocytes were resuspended and separated via Percoll gradient centrifugation. The interphases were collected, washed once with media, and spun down at 500 *g* for 7 min at 4°C prior to use in experiments.

PPs were rinsed in cold HBSS w/o Ca^2+^Mg^2+^, supplemented with EDTA (5 mM) and HEPES (5 mM), and incubated in the same solution for 10 min at 37°C with mild agitation. Epithelial remains were removed and PPs were mashed through a 70-µm cell strainer. Samples were washed once with FACS buffer (PBS, 5 mM EDTA, 2% FBS) and spun down at 500 *g* for 7 min at 4°C prior to the use in experiments.

MLNs were dissected and the surrounding fat was removed. MLNs were mashed through a 70-µm cell strainer. Samples were washed once with FACS buffer and spun down at 500 *g* for 7 min at 4°C prior to use in experiments.

Spleens were dissected and mashed through a 70-µm cell strainer. Samples were washed once with PBS and spun down at 500 *g* for 7 min at 4°C. The supernatant was removed and the samples were incubated in 1 ml of 1× red cell lysis (RCL) buffer for 3 min at RT. Samples were washed once with FACS buffer and spun down at 500 *g* for 7 min at 4°C prior to the use in experiments.

Femur and tibia were collected and the remaining soft tissues were removed from the bones. Bone marrow was flushed out using cold PBS and samples were washed once with PBS. The supernatant was removed and samples were incubated in 1 ml of 1x RCL buffer for 3 min at RT. Samples were washed once with FACS buffer and spun down at 500 *g* for 7 min at 4°C prior to the use in experiments.

### Fecal bacterial processing

Mouse fecal samples were collected in 1 ml of PBS in sterile Eppendorf tubes. Fecal weights were recorded. Samples were mechanically disrupted at 2,500 rpm for 3 min using a bead disruptor (without beads). Samples rested on ice for 5 min and filtered using a 70-µm filter. Filtered solutions were transferred into a new Eppendorf tube. Samples were spun at 50 *g* for 3 min and the supernatant was transferred to a new Eppendorf tube. Samples were washed twice with PBS prior to use.

### Microbial flow cytometry

Bacteria were resuspended in bacteria-blocking buffer (BBB: PBS, 20% [vol/vol] goat serum) and incubated at 4°C for 20 min. Samples were washed with bacteria staining buffer (BSB: PBS, 1% BSA, 40-μM filtered) and spun at 8,000 *g* for 4 min. Fecal bacterial samples from *Rag2*^*−/−*^ mice were incubated with 50 μl of serum for 30 min at 4°C and washed twice with BSB. Bacteria were stained with fluorophore-conjugated anti-IgA antibodies in BSB in the dark at 4°C for 30 min and washed twice with BSB at 8,000 *g* for 4 min. Samples were further stained with PBS-supplemented SytoBC (1:2,000) at 4°C for 10 min in the dark, washed twice, and resuspended in BSB for analysis. For the analysis of IgA coating on *T.mu*, caeca were harvested, opened, and the content resuspended in 10 ml of sterile PBS. Suspensions were spun at 500 *g* for 7 min at 4°C. The supernatant was discarded and the pellet was washed twice with sterile PBS. Protozoa were enriched through a Percoll gradient and the interphase was collected, washed, and filtered through a 70-µm cell strainer. *T.mu* were stained with fluorophore-conjugated anti-IgA antibodies in FACS buffer for 20 min on ice. Stained protozoa were washed and analyzed on a BD LSRFortessa X-20.

### Flow cytometry

#### Surface staining

Cells were washed once with FACS buffer and spun down at 500 *g* for 4 min at 4°C. For CXCR5 surface staining, samples were incubated with anti-CXCR5 in FACS buffer (1/50 – RT – 45 min). Samples were washed twice at 500 *g* for 4 min at 4°C and then incubated with corresponding surface antibody cocktails in FACS buffer at 4°C for 30 min. Samples were washed twice and resuspended in FACS buffer for analysis.

#### Intracellular cytokine staining

Harvested MLN cells were resuspended in complete RPMI supplemented with Brefeldin A solution (Biolegend) and cell activation cocktail (Biolegend) at 37°C for 4 h. After incubation, cells were washed with ice-cold FACS buffer and stained for surface markers as described above. Following another wash step, cells were fixed and permeabilized using the BD Cytofix/Cytoperm solutions. Intracellular staining for IL-17A was performed using anti-IL17A antibodies resuspended in BD PermBuffer for 20 min. Cells were washed with BD PermBuffer and analyzed on a BD LSRFortessa X-20.

#### Intranuclear staining

Following surface staining, intracellular staining was performed using the eBioscience Foxp3 kit following the manufacturer’s recommendations. Samples were resuspended in intracellular staining buffer analysis. Flow cytometry was performed on a BD LSRFortessa X-20 and analyzed using FlowJo (v10.1).

### Fluorescent activated cell sorting

#### Lymphocyte FACS

Lymphocyte sorting was performed on a BD FACSAria. Up to 5 million cells were sorted into 1 ml of sorting buffer (SB–50% FACS buffer–50% FBS) for downstream applications.

#### Fecal bacteria FACS

Fecal bacteria sorting was performed on a BD FACSAria. Up to 2 million cells were sorted into 1 ml of DNAzap (Invitrogen) for downstream applications.

### Immunofluorescence microscopy

#### Colon sample preparation

Following colon cleaning, samples were flushed with cold PBS. Colons were cut into four equal parts and placed in 2 ml of fixative solution (2% PFA - 20% sucrose - 1× PBS). Samples were fixed for 1 h at RT and transferred into PBS on ice for 1 h. Samples were incubated in 40% sucrose/PBS overnight at 4°C. Samples were embedded into OCT and stored at −80°C. Tissues were sectioned at 7 μm onto Fisherbrand Superfrost slides, and the slides were stored at −20°C.

#### IF staining procedure

Sections were brought to RT, circled with a PAP pen (Abcam), and air-dried for 15 min. Sections were then rehydrated three times with PBS for 5 min and blocked with blocking buffer (10% BSA - 0.1% TritonX – PBS) for 1 h at RT in a humidified chamber. Sections were washed and stained with anti-IgA FITC (clone 11-44-2, 1:100; Southern Biotech) in a blocking buffer in the dark for 30 min at RT. Stained sections were washed and mounted with Fluoroshield mounting media with DAPI (Abcam). Slides were stored in the dark at 4°C or visualized immediately on a ZEISS Axio Imager.

### CD4 T cell depletion

Mice were injected intraperitoneally (i.p.) with depleting anti-CD4 Ig 25 mg/kg (clone GK1.5; BioXcell) in sterile PBS for 3 days prior to *T.mu* colonization and with 15 mg/kg anti-CD4 Ig at D1, D7, and D14 after *T.mu* colonization.

### CD4^+^ T cell transfer

Live, CD45^+^, CD3^+^, and CD4^+^ splenic T cells were sorted on a BD FACSAria. Cells were resuspended in cold PBS, and 2 × 10^5^ T cells were injected i.v. into age- and sex-matched *Tcrb*^*−/−*^ mice. Two weeks after injection, mice were colonized with *T.mu*.

### OVA treatment and adoptive transfer of OTII^Tg^Thy1.1 cells

4 × 10^5^ CD4^+^ cells were sorted from the spleen of OTII^Tg^Thy1.1 mice and injected i.v. into *Tcrb*^*−/−*^ recipient mice previously colonized with *T.mu* or not. All mice received 1% OVA (Grade V; Millipore Sigma) via drinking water ad libitum for 10 days following injection. OVA-containing drinking water was freshly prepared and sterile filtered through a 0.22 µm filter every 2 days.

### FITC-dextran assay

Barrier integrity was assessed using FITC-Dextran. Groups of mice were either left untreated or were colonized with *T.mu*. 3 wk after colonization, mice were gavaged with 150 μl FITC-dextran (80 mg/ml) in PBS and removed from any food source for 4 h. Blood was collected through cardiac puncture and acid-citrate dextrose was added to the blood to a final concentration of 15% vol/vol. Serum was collected through centrifugation at 4°C, diluted, and analyzed on a SpectraMAX spectral reader at an excitation wavelength of 485 nm. Concentrations were calculated using a standard curve.

### ICOSL blockade

Mice were injected i.p. with blocking anti-ICOSL Ig at 25 mg/kg (HK5.3; BioXCell) 1 day prior to *T.mu* colonization. After colonization, mice received 25 mg/kg of anti-ICOSL Ig three times per week for the remainder of the experiments.

### Ovalbumin treatment

Three weeks after *T.mu* colonization, mice were offered 1% OVA (Grade V; Millipore Sigma) in sterile drinking water ad libitum. OVA-containing drinking water was freshly prepared and sterile-filtered through a 0.22-µm filter every 2 days for 3 wk.

### Bone marrow chimeras

B cell-deficient *µMT*^*−/−*^ mice received a dose of 1,300 cGy total body irradiation over two doses of 650 cGy, separated by a 12-h period. 4 h following the last dose, recipient mice were i.v.-injected with a total of 5 × 10^6^ bone marrow leukocytes. Mice were treated with streptomycin-containing drinking water (1 g/L) for the 2 wk after bone marrow injections. 10 wk after bone marrow injections, mice were colonized with *T.mu*.

### Total Ig ELISA

Mouse blood was collected and isolated using Microvette CV 300 Z serum separation tubes (SARSTEDT) according to the manufacturer’s protocol. 50 μl of anti-mouse Ig (1:2,000; SouthernBiotech) in PBS were added to 96-well high-bound Nunc MaxiSorp ELISA plates (BioLegend) and incubated at 4°C overnight. Plates were washed and blocked, and serum and fecal samples (1:100, 1:10) were added. Samples were incubated for 1 h at 37°C. Plates were washed and incubated with 50 μl of anti-mouse IgA-HRP (1:1,000) or anti-mouse IgG1, IgG3, IgG2b, or IgG3c (all 1:1,000) in assay buffer (SouthernBiotech). Plates were washed and developed using TMB substrate (BioLegend). The reaction was stopped using 50 μl of H_2_SO_4_. Plates were read by spectrophotometry (450 nm excitation) and absorbance values were interpolated based on a purified IgA or IgG standard curve.

### Anti-OVA IgA ELISA

96-well ELISA plates (BioLegend) were coated with 50 μl of Ovalbumin from chicken egg white (1:1,000 in 1x PBS; Sigma-Aldrich) at 4°C overnight. Plates were washed and blocked prior to incubation with 100 μl of serum (1:100 dilution) or fecal supernatant (1:10 dilution). Anti-OVA IgA standards (Chondrex) were run in parallel on each plate. Plates were then washed and incubated with 50 μl of anti-mouse IgA-HRP (1:1,000 in PBT; SouthernBiotech), and washed and developed using 50 μl of TMB substrate (BioLegend). The reaction was stopped using 50 μl of H_2_SO_4_ and the plates were read on a spectrophotometr (450 nm excitation), and absorbance values were interpolated based on a purified IgA standard curve.

### Anti-OVA and total IgA ELISPOT

96-well filter plates (Millipore) were coated with 50 μl of Ovalbumin from chicken egg white (1:1,000 in 1X PBS; Sigma-Aldrich) and incubated at 4°C overnight. Plates were washed and blocked and 100 μl of diluted cell samples in complete RPMI was added to the plates and titrated. Plates were incubated overnight at 37°C in a tissue culture chamber. The following day, plates were washed and 100 μl of anti-mouse IgA-HRP (SouthernBiotech) was added followed by incubation at 37°C for 2 h. Plates were washed two times with PBST and incubated with mild shaking at RT for 10 min during the third wash. Plates were then washed twice with PBST. 50 μl of AEC buffer (SK-4200, in distilled water; Vector laboratories) was added to each well, followed by distilled water to stop the reaction after 9 min. Plates were placed in the dark for drying overnight. The following day, red-colored spots on each well’s membrane were counted as one ASC.

### DNA isolation for whole fecal samples

DNA isolation was performed using the QIAQuick qPCR purification kit (QIAGEN) with modifications. Fecal weights were measured, and flash-frozen pellets were defrosted on ice for 10 min. SDS buffer (8 g SDS – 30 ml H_2_O – 0.22 μm filtered) and DNA buffer A (20 mM Tris pH 8 – 2 mM EDTA – 200 mM NaCl) were prepared. SDS buffer and DNA buffer A were combined in a 1:1.41 ratio to prepare SDS-A buffer. Samples were transferred to cryogenic 2 ml tubes and 400 μl zirconium beads, 550 μl of phenol–chloroform, and 482 μl of SDS-A buffer were mixed. Samples were bead-beaten at 1,800 *g* for 5 min. Samples were spun at 1,800 *g* for 5 min, and the supernatant was transferred into a QIAGEN PCR purification plate containing 650 μl of PM buffer (QIAQuick qPCR kit). Samples were washed twice with 900 μl of PE buffer (QIAQuick qPCR kit). Samples were spun at 1,800 *g* for 5 min to remove trace ethanol. To elute DNA, 100 μl of DNAse/RNAse-free H_2_O was added to each well. Samples were incubated at RT for 5 min and spun at 1,800 *g* for 2 min to collect DNA. DNA concentrations were quantified using the Qbit Quant-iT kit.

### RNA isolation

Whole colonic tissue or purified *T.mu* was resuspended in 500 μl of TRIzol and the RNA was isolated using the phenol/chloroform extraction. The recovered RNA was resuspended in 50 μl of RNase-free water and stored at −80°C. For FACS-sorted cells, RNA was purified in accordance to the RNeasy kit (QIAGEN). RNA concentration was quantified using a NanoDrop.

### Reverse transcription of mRNA

Total mRNA was converted to cDNA using the High-Capacity RNA-to-cDNA kit (Applied Biosystems). In brief, purified mRNA was mixed with 2× Reverse Transcription Buffer Mix, 20× Reverse Transcription Enzyme Mix, and nuclease-free H_2_O. The thermal cycling conditions were conducted as follows: 37°C for 60 min, 95°C for 5 min, and infinite hold at 4°C. Controls for reverse transcription included a non-template control, no enzyme control, and water-only control.

### Digital droplet PCR (ddPCR)

ddPCR was performed following the QX200TM ddPCRTM EvaGreen Supermix kit instructions (Bio-Rad). In brief, 50 ng of DNA template per reaction was mixed in nuclease-free ddPCR plates with 2× QX200TM ddPCRTM EvaGreen Supermix, 100 nM of forward/reverse primer, 1 μl of diluted restriction enzyme and nuclease-free water. Oil droplet generation was performed in a QX200 AutoDG Droplet Digital PCR System (Bio-Rad) using the QX200 Droplet Generation oil for EvaGreen (cat #1864005; Bio-Rad). The thermal cycling conditions were conducted as follows: Enzyme inactivation at 95°C for 5 min (1 cycle)–denaturation at 95°C for 30 s, annealing/extension at 60°C for 60 s (40 cycles)–signal stabilization at 4°C for 5 min followed by 90°C for 5 min (1 cycle)–hold at 4°C. The ramp rate between cycling steps was set at 2°C/s. Samples were analyzed using the QX200 Droplet Reader (Bio-Rad) and analyzed using the QuantaSoftTM software (Bio-Rad). Values were reported as absolute concentration as copies/μl.

### Quantitative real-time PCR (qPCR)

qPCR was performed following the PowerTrack SYBR Green Master Mix kit instructions (Applied Biosystems). In brief, 10 ng of DNA per reaction was mixed in nuclease-free qPCR plates with 2X PowerTrack Master Mix, 100 nM of forward/reverse primer, and nuclease-free water. The thermal cycling conditions were conducted as follows: Enzyme inactivation at 95°C for 2 min (1 cycle)–denaturation at 95°C for 15 s, annealing/extension at 60°C for 60 s (40 cycles). Controls for qPCR included a non-template control, no master mix control, and water-only control. Relative expression was calculated using the Δ-Δ Ct method.

### 16S rDNA analysis

Purified DNA from mouse fecal pellets or sorted cells was subjected to 16S variable region 4 PCR amplification using barcoded 515F and 806R primers (Caporaso et al., 2012) and the KAPA2G Robust HotStart ReadyMix (KAPA Biosystems), with the following cycling conditions: 95°C for 3 min, 24× cycles of 95°C for 15 s, 50°C for 15 s and 72°C for 15 s, followed by a 5-min 72°C extension. All reactions were performed in triplicate and pooled. The amplicon libraries were combined and purified using Ampure XP beads, and sequenced at the Center for the Analysis of Genome Evolution and Function (CAGEF) at the University of Toronto on an Illumina MiSeq using V2 (150 bp *×*2) chemistry, according to manufacturer instructions (Illumina). Amplicon sequences were quality-filtered and classified using the UNOISE pipeline, available through USEARCH v11.0.667 and vsearch v2.10.4 (Edgar and Flyvbjerg, 2015; Edgar, 2010, 2013; Rognes et al., 2016). Briefly, low-quality bases were trimmed using Cutadapt v.1.18, and the read pairs were merged using vsearch with assemble lengths set between 243 and 263. Amplicons were denoised and filtered for chimeras and clustered to 99% identity OTUs. Taxonomies were assigned using SINTAX and the Ribosomal Database Project (RDP) database version 16, with a minimum confidence cutoff of 0.8 (Wang et al., 2007). A phylogenetic tree was generated using FastTree (Price et al., 2009).

Compositional and diversity analyses of bacterial data were carried out using R-packages Phyloseq 1.26.1 and vegan v.2.5. Alpha diversity metrics (observed OTUs and the Shannon Diversity Index) were determined from averages of 100 independent datasets rarefied to the minimum sample read depth (19,427), and group differences were evaluated using the generalized linear models (glm) function in R. Beta diversities (compositional differences among samples) were calculated based on Bray–Curtis dissimilarity scores using rarefied OTU data (filtered for OTUs with minimum five reads) and tested for group differences using the adonis function in vegan. Differential taxon abundance among groups was determined with DESeq2 v.1.22.2. Heatmaps of bacterial abundance were generated using variance stabilized counts with the package pheatmap v1.0.12.

### Statistical analysis

Statistics were performed using Prism 7 software. Error bars are presented using the standard of error of the mean (SEM). Student’s *t* test, Mann–Whitney, one-way ANOVA, and two-way ANOVA tests using Prism 7 were used to determine statistical significance where appropriate. Significance was indicated as follows: * = P < 0.05, ** = P < 0.01, *** = P < 0.001, **** = P < 0.0001, ns = not significant. Antibodies are listed in Table 1. Reagents and resources are listed in the Table 2. Primers are listed in Table 3.

**Table 1.** Antibodies

Fluorophore	Antibody	Clone	Company	Reference #
APC	Rat anti-mouse B220 (CD45R)	RA3-6B2	BioLegend	103212
APC	Rat anti-mouse PD-1 (CD279)	29F.1A12	BioLegend	135210
AF647	Goat anti-mouse IgA	N/A	Southern Biotech	1040-31
eF450	Rat anti-mouse CD3	17A2	eBioscience	48-0032-82
eF450	Rat anti-mouse MHC-II (I-A/I-E)	M5/114.15.2	BioLegend	107607
FITC	Rat anti-mouse CD40L (CD154)	SA047C3	BioLegend	157005
FITC	Rat anti-mouse CD4	GK1.5	BioLegend	100406
FITC	Rat anti-mouse CD40L (CD154)	SA047C3	BioLegend	157006
FITC	Anti-mouse FAS (CD95)	SA367H8	BioLegend	152606
FITC	Goat anti-mouse IgA	N/A	Southern Biotech	1165-02
FITC	Rat anti-mouse Ki-67	16A8	BioLegend	652410
FITC	Rat anti-mouse/rat XCR1	ZET	BioLegend	148210
FITC	SYTOBC green fluorescent nucleic acid stain	N/A	ThermoFisher	S34855
APC	Rat anti-mouse B220	RA3-6B2	BioLegend	103231
APC	Rat anti-mouse PD-1	RMP1-30	BioLegend	109111
APC	Rat anti-mouse CD40	3/23	BioLegend	124612
APC Cy7	Anti-mouse CD45.2	104	BioLegend	109824
PE	Rat anti-mouse CD197 (CCR7)	4B12	BioLegend	120106
PE	Rat anti-mouse CD138	281-2	BioLegend	142504
PE	Hamster anti-mouse CD154	MR1	BioLegend	106505
PE	Rat anti-mouse GL-7	GL7	BioLegend	144608
PE	Rat anti-mouse Foxp3	MF-14	BioLegend	126404
PE	Rat anti-mouse CCR7	4B12	BioLegend	120106
PE	Rat anti-mouse ICOSL	HK5.3	BioLegend	107405
PE Cy7	Anti-mouse/human CD11b	M1/70	BioLegend	101216
PE Cy7	Hamster anti-mouse ICOS	C398.4A	BioLegend	313520
PE Cy7	Rat anti-mouse CD4	GK1.5	BioLegend	100422
PerCP Cy5.5	Rat anti-mouse IgM	RMM-1	BioLegend	406512
PerCP e710	Rat anti-mouse CXCR5	SPRCL5	eBioscience	46-7185-82
BV 605	Rat anti-mouse B220	RA3-6B2	BioLegend	103243
eFluor 506	Fixable viability	N/A	eBioscience	65-0866-14
HRP	Goat anti-mouse IgA	N/A	Southern Biotech	1040-05
N/A	InVivoMAb anti-mouse ICOSL	HK5.3,	BioXCell	BE0028
N/A	InVivoMAb anti-mouse CD4	GK1.5	BioXCell	BE0003-1

**Table 2.** Reagents and resources

Reagent or resource	Source	Identifier
Dulbecco’s phosphate buffered saline (1×)	Sigma-Aldrich	BSS-1005-B
Dulbecco’s phosphate buffered saline (10×)	Sigma-Aldrich	6506-OP
Dulbecco’s phosphate buffered saline (1×) w/o CA^+^ & Mg^++^	Sigma-Aldrich	BSS-1006
Hanks’ balanced salt solution w/ CA^++^ & Mg^++^ (1×)	GIBCO	14175095
Bovine serum albumin	Millipore sigma	B6917
GlutaMAX	GIBCO	35050061
Sodium pyruvate	GIBCO	11360070
MEM non-essential amino acids solution	GIBCO	11140050
2-Mercaptoethanol	Sigma-Aldrich	M6250
Red cell lysis buffer	Biolegend	420301
Percoll	Sigma-Aldrich	P1644
Dulbecco’s modified Eagle medium	Gibco	11995-065
RPMI-1640	Sigma-Aldrich	R8758-500 ML
DNAse	Sigma-Aldrich	DN-25
Collagenase	Sigma-Aldrich	C5138-1G
EDTA pH 8.0, 0.5M	BioShop	EDT111.500
HEPES	Gibco	15630-080
Fetal bovine serum	Gibco	26140079
Foxp3 transcription factor staining buffer set	eBioscience	00-5523-00
DNAZap	Invitrogen	AM9890
Fischerbrand superfrost plus microscope slides	Fischerscientific	12-550-15
TritonX	Sigma-Aldrich	T9284
Streptomycin sulfate salt	Sigma-Aldrich	S6501
Penicillin-streptomycin	Sigma-Aldrich	P4333
Fluoroshield mounting media	Sigma-Aldrich	F6182
Fluoroshield mounting media with DAPI	Sigma-Aldrich	F6057
Microvette CB 300Z blood collection capillary tube	Sarstedt	16.440.100
Nunc MaxiSorp ELISA plates, uncoated	BioLegend	423501
Mouse Anti-OVA IgA antibody assay kit	Chondrex	3018
TMB substrate	Biolegend	422101
Sulfuric acid (5.0N Solution)	Bioshop	SUL500.1
Tween20	Bioshop	TWN510.500
UltraPure 1M Tric-HCL pH 7.5	Invitrogen	15567-027
Ovalumin grade V	Millipore sigma	A5503
Zirconium beads	BioSpec	11079101z
QIAquick qPCR purification kit	QIAGEN	28104
TRI reagent (Trizol)	Sigma-Aldrich	T9424
RNeasy mini kit	QIAGEN	74106
High-capacity RNA-to-cDNA	Applied Biosystems	4387406
QX200 ddPCR EvaGreen supermix	Bio-Rad	1864034
QX200 droplet Generation oil for EvaGreen	Bio-Rad	1864005
PowerTrack SYBR green MM	ThermoFisher	A46012
QIAamp DNA microbiome kit	QIAGEN	51704
Streptomycin sulfate salt	Sigma-Aldrich	S6501-50 G
Phenol-chloroform	Ambion	AM9732
Chloroform	Caledon	3000-1-10

**Table 3.** Primers

Gene	Forward primer	Reverse primer
*Tnfa*	5′-CCT​GTA​GCC​CAC​GTC​GTA​G-3′	5′-GGG​AGT​AGA​CAA​GGT​ACA​ACC​C-3′
*Il5*	5′-CTG​CAA​GAG​ACT​TCC​ATC​CA-3′	5′-AGT​GGT​ATA​GAC​AGG​TCT​GTT​GG-3′
*Il6*	5′-GCA​ATG​AGA​CGA​TGA​GGC​TTC-3′	5′-GCC​CCT​GAA​AGA​TTT​CTC​CAA​TG-3′
*Cxcl13*	5′-ATA​TGT​GTG​AAT​CCT​CGT​GCC​A-3′	5′-GGG​AGT​TGA​AGA​CAG​ACT​TTT​GC-3′
*Tgfb*	5′-CTT​CAA​TAC​GTC​AGA​CAT​TCG​GG-3′	5′-GTA​ACG​CCA​GGA​ATT​GTT​GCT​A-3′
*Nos2*	5′-GGA​GTG​ACG​GCA​AAC​ATG​ACT-3′	5′-TCG​ATG​CAC​AAC​TGG​GTG​AAC-3′

### Online supplemental material

Fig. S1 shows flow cytometry, ELISA, and gene expression data relating to Fig. 1 and Fig. 2. Additionally, Fig. S1 provides supporting data emphasizing the claims resulting from data in Fig. 2, A–D. Fig. S2 shows data that relates to Fig. 2, C–J, and supports in vivo observations. Fig. S3 shows flow cytometry data supporting the experiments described in Fig. 3.

## Supplementary Material

SourceData F3 is the source file for Fig. 3.

## Data Availability

Sequence data were deposited to the NCBI SRA, BioProject ID PRJNA1171466.
