# Supplementary figures and images for: The protozoan commensal Tritrichomonas musculis is a natural adjuvant for mucosal IgA
Source: J Exp Med. 2024 Nov 13;221(12):e20221727. doi: 10.1084/jem.20221727 (PMC11561467; doi:10.1084/jem.20221727)

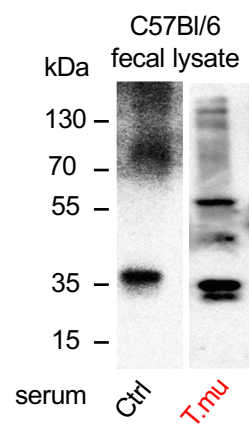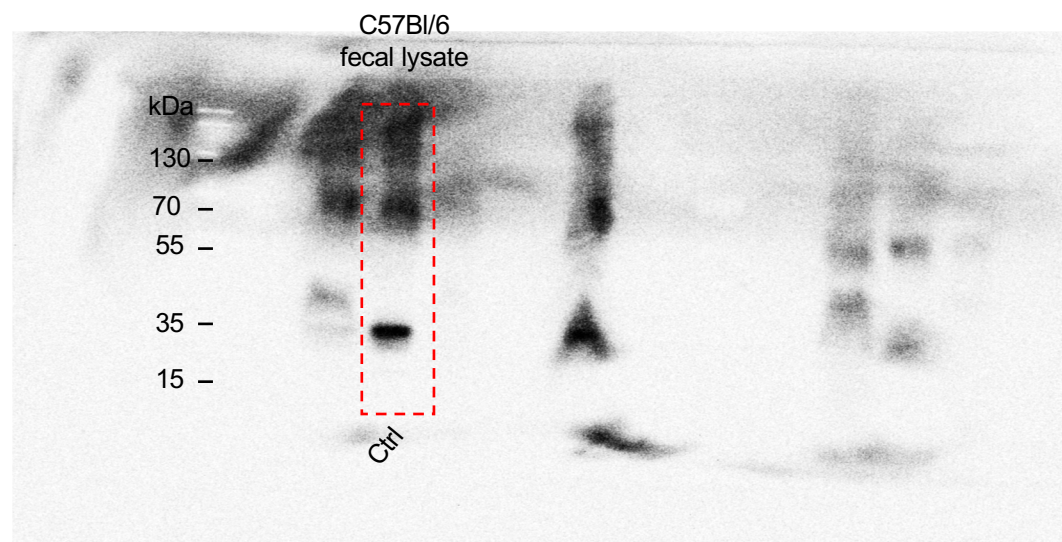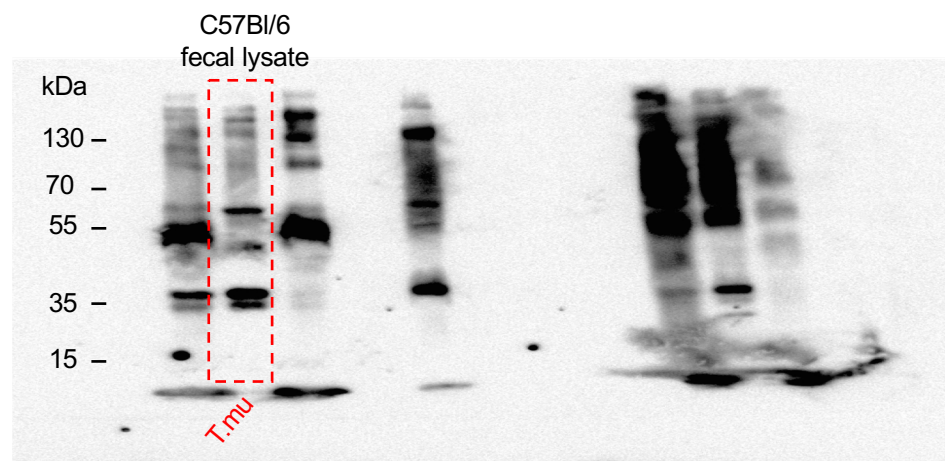

Supplement: SourceData F3 — is the source file for Fig. 3. [file JEM_20221727_SourceDataF3.pdf]
